# Long‐Term Temporal Change of Small Mammal Composition and Abundance in the Food Intake of the Western Barn Owl ( *Tyto alba* ), Comparing Two Different Lowland Mesoregions

**DOI:** 10.1002/ece3.74309

**Published:** 2026-09-11

**Authors:** Győző F. Horváth, Gábor Takács, Dániel Molnár, Adrienn Horváth

**Affiliations:** ^1^ Department of Ecology University of Pécs Pécs Hungary; ^2^ Fertő‐Hanság National Park Directorate Sarród Hungary; ^3^ Danube‐Drava National Park Directorate Pécs Hungary

**Keywords:** alpha diversity, community dynamic characteristics, pellet analysis, rodents, shrews

## Abstract

The change in the composition and diversity of small mammal assemblages, as well as the diet composition of the Western Barn Owl, has long been researched around the world; however, there are few long‐term studies in Hungary. The object of this study is to evaluate and compare the changes of the small mammal assemblages between two lowland areas. We analyzed Western Barn Owl pellets collected between 2000 and 2023 to investigate the long‐term temporal change of small mammal composition and abundance in the owls' diet. We investigated the regional differences in small mammal community structure by iNEXT, PERMANOVA, and RAC analysis. Generalized additive models (GAMs) and generalized additive mixed models (GAMMs) were applied to analyze the temporal change of diversity and community dynamic characteristics (turnover, rank change, and rank shift) of small mammal assemblages and the time‐ and area‐dependent abundance change of the consumed prey taxa, as well as the impact of weather conditions. The abundance of prey species was significantly different between the two areas. Based on our results, the diversity indices of the Western Barn Owls' diet were significantly higher in the Győr basin than in the Drava floodplain. Our GAM and GAMM results confirmed that there were significant temporal and spatial changes in the diversity indices, the rank shift, and the abundance of prey taxa. These findings suggest that prey occurrence and small mammal community structure can vary depending on local environmental factors, and the examination of Western Barn Owls' food composition can contribute to understanding the change of these assemblages under changing conditions.

## Graphical abstract

The object of this study is to evaluate and compare the changes of the small mammal assemblages between two lowland mesoregions in Hungary based on Western Barn Owl pellet analysis. Prey composition, diversity, and community dynamics differed markedly between the two lowland regions, indicating that local landscape characteristics and weather conditions jointly shape the prey assemblages available to hunting owls. Moreover, the effects of weather variables were taxon‐specific and differed between regions, highlighting that the responses of small mammals to climatic conditions depend on local environmental context.

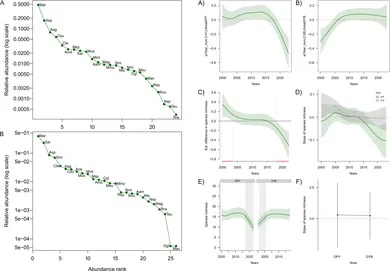

## 1 Introduction

The Western Barn Owl ( *Tyto alba* Scopoli, 1769) is a medium‐sized, nocturnal cosmopolitan owl species (Bunn et al. 1982; Roulin et al. 2012; Shawyer 1998; Taylor 2004); its range extends to temperate, subtropical, and tropical regions. This synanthropic owl mainly occurs in open, semi‐natural habitats and mosaic habitats in agricultural areas in the Palearctic region in Europe (Askew et al. 2007; de Bruijn 1994; Frey et al. 2011; Horváth et al. 2018), as well as North America (Hindmarch et al. 2012; Kross et al. 2016; Looman et al. 1996; Marti 1988, 2010) and South America (Bellocq 2000; Leveau et al. 2006; Trejo and Lambertucci 2007) in temperate areas. The landscape‐focused habitat use of Western Barn Owls has also been documented in the Mediterranean and semiarid subtropical areas (Andrade and Monjeau 2014; Avery 1999; Charter et al. 2012; Martínez and López 1999; Martínez and Zuberogoitia 2004; Paspali et al. 2013). The Western Barn Owl is characterized by a human‐associated life history and nests typically in different man‐made structures (e.g., church towers, belfries, barns, warehouses, stables), which eases pellet collection (Taylor 2004). This owl species mainly preys on terrestrial small mammals (voles, mice, and shrews) (Askew et al. 2007; Brown et al. 1982; Jaksić et al. 1982; Marti et al. 2005; Mikkola 1983; Taylor 2004; Tores et al. 2005), but based on the investigations of their food composition in different biogeographical regions, there is no consensus on whether this synanthropic owl species is a generalist (Baudrot et al. 2016; Debus et al. 2004; Herrera 1974; Love et al. 2000; Vargas et al. 2002), an opportunist (Bernard et al. 2010; Love et al. 2000; Meek et al. 2012; Mikkola 1976; Tores et al. 2005), or a small mammal specialist (Charter et al. 2018; Hindmarch and Elliott 2015; Marti et al. 2005; Milana et al. 2016; Taylor 2004; Trejo and Lambertucci 2007). In addition, there is a close relationship between the Western Barn Owl's niche breadth and research intensity. Besides the easy collection and analysis of its pellets, the food niche breadth of this owl species is larger compared to other owl and bird of prey species, and its niche breadth directly reflects the structure of the small mammal community in a given area (Andrade et al. 2016; Avenant 2005; Everett et al. 1992; Heisler et al. 2016; Torre et al. 2004).

The phylogeny of barn owls (Tytonidae) is an important factor that would explain the different food intake of barn owls from sympatric strigid owl species (Comay and Dayan 2018). The early Miocene witnessed rapid diversification of strigid owls, outcompeting the ancient barn owls due to competition (Ballman 1969; Mlíkovský 1998, 1999; Mourer‐Chauviré 1999). In the Middle and Late Miocene, open habitats expanded (Spriggs et al. 2014; Strömberg 2005), which favored the adaptive radiation of small mammals (Badgley et al. 2008; Sabol et al. 2021). The modern genus Tyto has adapted to exploit the small mammal biomass of these open habitats (Burton 1984). The modern tytonid owls developed specialized morphology (e.g., asymmetrical ears (Kaup 1862; Norberg 2002; Payne 1971; Ridgway 1914; Schwartzkopff 1962); heart‐shaped facial disc and auricular feathers (Coles and Guppy 1988; Koch and Wagner 2002; Payne 1971; von Campenhausen and Wagner 2006); and slow, low‐altitude flight (Bachmann et al. 2011; Schalcher et al. 2024; Usherwood et al. 2014)) to make it easier to hunt terrestrial rodents in open fields. To understand these mechanisms and the specialization of the Western Barn Owl, paleontological research would be necessary that clarifies its prey selection and food intake from an evolutionary perspective (Comay and Dayan 2018). Prey selection and actual food intake depend on several factors. One of the most important is size‐based prey selection, according to which the body size of the prey increases with the body size of the predator, and several studies confirmed this mechanism about the Western Barn Owl (Gotta and Pigozzi 1997; Hardy 1977; Marti 1973; Pokharel et al. 2021; Taylor 2004; Yom‐Tov and Wool 1997). However, others found contradictory results, which means that Western Barn Owl hunts lighter or heavier prey (Castro and Jaksic 1995; Derting and Cranford 1989; Dickman et al. 1991; Kotler et al. 1988; Marks and Marti 1984; Trejo and Guthmann 2003). These findings may be due to the fact that the food intake of owls also depends on, for example, prey availability (Andrade et al. 2016; Heisler et al. 2016; Milana et al. 2016); encounter rate (Taylor 2009); taxonomic identity (Comay and Dayan 2018); habitat and environmental factors (Marti 1988; Onduso et al. 2025; Paspali et al. 2023; Romano et al. 2020); or handling mechanism (Ille 1991; Vassallo et al. 1994). Besides these, the multi‐species functional response (MSFR) and prey switching mechanism relating to MSFR as an adaptation of owls' foraging strategies need to be considered (Baudrot et al. 2016; Giraudoux et al. 2020; Thomsen et al. 2018), which can affect the dynamics of the prey populations (Baudrot et al. 2016).

The collection and analysis of the Western Barn Owl's pellets is primarily a feeding ecological approach because it allows for the comparison of seasonal (Bosé and Guidali 2001; González‐Fischer et al. 2011, 2012; Latková 2008; Paspali et al. 2013; Smith et al. 1972) and long‐term changes in prey consumption (Love et al. 2000; Lyman 2012; Marti 1988, 2010) over time or along different gradients (Hindmarch and Elliott 2015; Leveau et al. 2006; Teta et al. 2012; Travaini et al. 1997; Trejo and Lambertucci 2007), as well as the detection of differences in food composition between geographical regions (Barbosa et al. 1992; Milana et al. 2016) and landscapes with different utilization patterns and habitat structures (Battisti et al. 2020; Bontzorlos et al. 2005; Charter et al. 2009; Horváth et al. 2018, 2022; Milchev 2015; Moysi et al. 2018; Rodríguez and Peris 2007; Veselovský et al. 2017).

Several case studies focused primarily on assessing the composition of small mammal assemblages and estimating their species richness and diversity, some of which only used pellet analysis (Balestrieri et al. 2019; González‐Fischer et al. 2012; Horváth et al. 2022; Massa et al. 2014; Milana et al. 2019; Millán de la Peña et al. 2003; Stefke and Landler 2020; Torre et al. 2015; Vigués et al. 2018), while other studies used live trapping sampling in addition to the indirect method (Andrade and Monjeau 2014; Avenant 2005; Bernard et al. 2010; Heisler et al. 2016; Rocha et al. 2011). Furthermore, numerous studies have used data from pellet analysis to examine the relationships between weather and climate variation and changes in the community composition of small mammals (Avery 1999; Szpunar et al. 2008; Thiam et al. 2008; Heisler et al. 2014; Torre et al. 2015).

Several long‐term Western Barn Owl pellet analyses have revealed trends in changes in the abundance of small mammals, both at the level of the species and taxonomic or functional groups (e.g., Love et al. 2000; Milana et al. 2018; Stefke and Landler 2020; Vigués et al. 2018). An earlier study of Clark Jr. and Bunck (1991) evaluated the data of seven decades, showing an increase in shrews proportional to the amount of precipitation and a parallel decrease in rodents. This study pointed out that human interventions have an effect on landscape structure, and its effect changed the availability of small mammals, which resulted in a change in the dietary composition of this owl species. Based on a Western Barn Owl food analysis covering 40 years, the years proved to be a better predictor than the different sample sites, but this effect was not significant in the change of prey richness; however, an increasing trend of the dominance index, while a decreasing trend of the Shannon diversity, was described as a function of time (Milana et al. 2018). In an area adjacent to North‐West Hungary in a biodiversity hotspot in Austria, relationships between the local climate influence and seasonal cyclic population fluctuation of small mammals as prey of Western Barn Owls were detected. In the case of the majority of species, increased precipitation and decreased temperature led to an increased abundance overall (Stefke and Landler 2020). Love et al. (2000) investigated long‐term changes in the feeding habits of British Western Barn Owls, highlighting the effects of intensive agricultural land use, which suggests that changes in the proportion of prey species, particularly rodents, can be linked to environmental changes. The impact of intensive agricultural cultivation has also been described in other areas. Agriculture negatively affects rare and habitat specialist species, while it favors the spread of habitat generalist species. In addition, agricultural activities affect species frequency (Millán de la Peña et al. 2003). Regarding the long‐term human‐induced landscape changes, the abundance of open guild decreased, while the abundance of forest and urban guilds increased in relation to the transformation of land use, which included a decrease in open habitats and an increase in forested and urban areas (Vigués et al. 2018).

In Hungary, small mammals have been monitored within the framework of the Hungarian Biodiversity Monitoring System (HBMS) since 2000, where a subproject investigates the distribution and abundance change of small mammal species (Csorba and Pecsenye 1997; Fodor et al. 2007; Horváth et al. 2019). Thus, we have had a long‐term dataset to evaluate and compare the change of small mammal assemblages in different landscapes. In our earlier studies of Western Barn Owl pellet analysis, we demonstrated that the common vole was detected as the main prey, and among Murid taxa, the *Apodemus* spp. was demonstrated as an important alternative prey of Western Barn Owls in the case of the Drava floodplain. In contrast, in addition to the higher proportion of common vole consumption, the Sorex species were identified as the main alternative prey in the Győr basin (Horváth et al. 2020, 2022; Horváth, Maurer, and Horváth 2023; Horváth and Horváth 2024). More studies in several European countries have confirmed that the common vole is the main prey (e.g., Bernard et al. 2010; Frey et al. 2011; Kitowski 2013; Petrovici et al. 2013; Szép et al. 2019; Veselovský et al. 2017) and wood mice (*Apodemus*) are a potential alternative prey (Achille et al. 2024; Bontzorlos et al. 2005; Pezzo and Morimando 1995; Rodríguez and Peris 2007) in the diet of Western Barn Owls.

The object of the present study is to evaluate the regional differences in small mammal community structure, the temporal trends in small mammal prey diversity, the temporal dynamics of prey community structure, and the species‐specific temporal responses and environmental drivers of small mammal abundance change in two different lowland areas.

## 2 Material and Methods

### 2.1 Study Areas and Sample Collection

**FIGURE 1:**
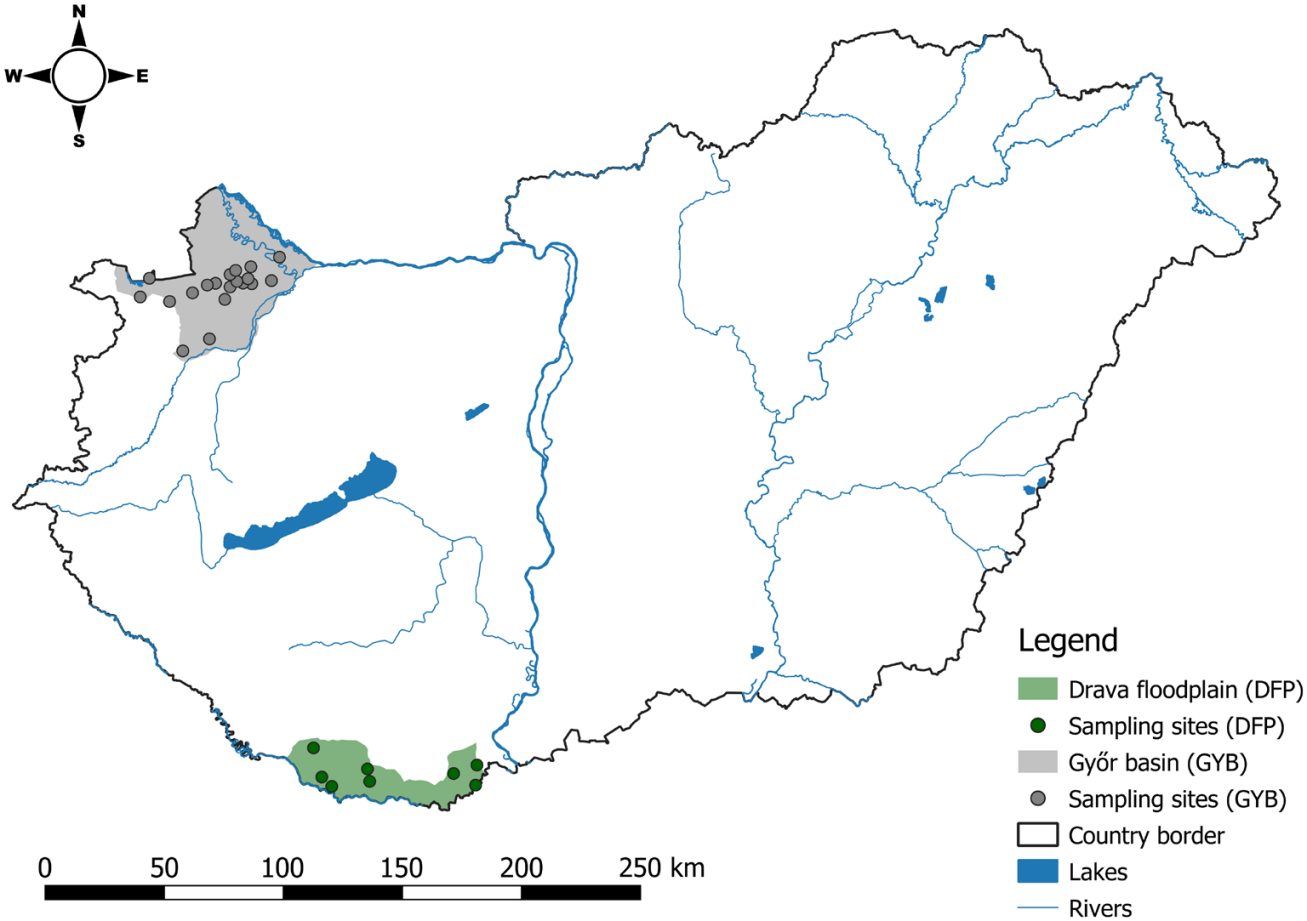
The study area in the DFP and in the GYB, showing the sampling sites.

The study was conducted in two mesoregions (later as area) in Hungary (Figure 1). The Drava floodplain [DFP] (45°87′ N, 18°08′ E) is situated in the southeastern part of the Transdanubian region, while the Győr basin [GYB] (47°69′ N, 17°24′ E) is located in the northwestern part of the Transdanubian region, in South and North Hungary, respectively. The area size of the DFP is 1300 km^2^, located along the Drava River within the territory of the Danube‐Drava National Park. The typical habitats of this landscape are 10–15 km^2^ in each place, with wide floodplains accompanied by backwaters and gallery forests. The climate of the DFP is moderately warm and moderately humid. The number of sunny hours is 2000–2080; the annual amount of precipitation is 630–720 mm (the western part is the wettest), and the average annual temperature is 10.4°C–10.6°C. In terms of wind conditions, it is characterized by moderate currents. The landscape structure of the DFP is fundamentally shaped by the Drava River. The landscape is highly mosaic and fragmented, where, in addition to natural elements (e.g., floodplain forests and oxbow lakes), agricultural areas can also be found. Agricultural cultivation is not characterized by the classic, homogeneous, and intensive large‐scale field structure. Instead, the fields are considerably more fragmented, with the region being characterized by medium or mixed parcel sizes. The GYB is located within the territory of the Fertő‐Hanság National Park, which is separated from Slovakia by the Danube as a natural border from the north, while it is bordered by Austria from the west. In the GYB, the average number of hours of sunshine is 1700–1900, the annual amount of precipitation is 650–750 mm, and the average annual temperature is 9°C–10°C. This region is characterized by a strong westerly‐north‐westerly air movement. The landscape structure of the GYB is determined by the alluvial deposition of the Danube, Rába, and Marcal rivers. In the Szigetköz, numerous river branches and floodplain forests are typical. Conversely, the Moson Plains and the Rábaköz are dominated by treeless agricultural areas. The Hanság and the immediate vicinity of Lake Fertő are characterized by reed beds and wet meadows of a formerly drained and canalized marshland. In contrast to the DFP, agricultural cultivation in the GYB is explicitly characterized by an industrial, intensive, large‐scale field structure.

Western Barn Owl pellets collected between 2000 and 2023 were used for the analysis. Due to the vast difference in the number of Western Barn Owl breeding pairs, in the first step, eight nesting sites from the DFP were selected for the analysis by randomization procedure. In the case of the GYB, we used data from those locations where there were samples for at least 2 years; thus, samples from 19 nesting sites were included in this study (Figure 1). A total of 8842 and 4336 pellets from the DFP and GYB were analyzed, respectively. In the latter area, pellet collections are less frequent, so the samples contain a large amount of nest and roost debris. Thus, despite the different amount of pellets, the minimum number of individuals in the samples was balanced between the two areas.

Pellets were processed using the dry technique because it has many advantages (e.g., there is no need for special equipment, solvent, or work area; it is more appropriate for immediate identification and educational purposes). Small mammals and other prey items were identified to the lowest possible taxonomical level based on skeletal parameters (skull, mandible and teeth, bill, and postcranial skeleton) (Kessler 2015; März 1972; Schäfer 1932; Yalden 1977; Yalden and Morris 1990). Due to the difficulty in identifying *Apodemus* species, we also used two larger prey categories (*Apodemus* spp., “unidentified Apodemus”), as described in our previous articles (Horváth et al. 2018, 2020, 2022; Horváth, Mánfai, and Horváth 2023; Horváth, Maurer, and Horváth 2023, Horváth and Horváth 2024).

The minimum number of individuals (MNI) of small mammal prey items was calculated by counting the same anatomical parts of bones (Horváth et al. 2022; Torre et al. 2015; Tulis et al. 2015). The percent frequency of occurrence (MNI%) was derived for the total number of prey found in all pellets in all different localities and samples. To improve the transparency and reproducibility of the dataset, the annual sampling effort for each study area (sample size, number of pellets, and total MNI) is presented in Appendix S1, while annual MNI values and percentage frequencies of the taxa included in the statistical analyses are provided in Appendix S2.

### 2.2 Statistical Analysis

Pooled MNI data of small mammal prey taxa were summarized in Table 1, separating the two investigated areas. From these MNI datasets, the relative abundance distribution of the highlighted main and alternative prey small mammals was tested based on the prop test, which was applied with the command prop.test in the package “stats” (v. 4.5.0; R Core Team 2024).

**TABLE 1:** Diet composition of the Western Barn Owl in the two investigated areas (MNI: Minimum number of individuals, %MNI: Percentage frequency of occurrence) (Author's Work).

Area	Drava floodplain		Győr basin	
Taxa [abbreviation]	MNI	%MNI	MNI	%MNI
Eulipotyphla				
Talpidae				
*Talpa europaea* [Teu]	12	0.06	13	0.07
Soricidae				
*Sorex araneus* [Sar]	471	2.30	4040	22.65
*Sorex minutus* [Smi]	179	0.88	1228	6.88
*Neomys fodiens* [Nfo]	155	0.76	50	0.28
*Neomys milleri* [Nmi]	136	0.67	74	0.41
*Neomys* spp. [Nsp]	14	0.07	36	0.20
*Crocidura suaveolens* [Csu]	1168	5.72	476	2.67
*Crocidura leucodon* [Cle]	672	3.29	627	3.51
Rodentia				
Cricetidae				
*Clethrionomys glareolus* [Cgl]	124	0.61	190	1.07
*Microtus agrestis* [Mag]	189	0.92	29	0.16
*Microtus arvalis* [Mar]	9659	47.26	6892	38.63
*Microtus subterraneus* [Msu]	113	0.55	156	0.87
*Alexandromys oeconomus* [Aoe]	0	0.00	398	2.23
*Arvicola amphibius* [Aam]	222	1.09	65	0.36
Muridae				
*Rattus norvegicus* [Rno]	31	0.15	68	0.38
*Rattus rattus* [Rra]	7	0.03	19	0.11
*Rattus* spp. [Rsp]	46	0.23	75	0.42
*Apodemus agrarius* [Aag]	1588	7.77	495	2.77
*Apodemus* spp. [Asp]	3453	19.90	1513	8.48
*Apodemus* indet. [Aind]	521	2.55	206	1.15
*Micromys minutus* [Mmi]	273	1.34	328	1.84
*Mus spicilegus* [Msp]	509	2.49	217	1.22
*Mus musculus* [Mmu]	185	0.91	153	0.86
*Mus* spp. [Mus]	444	2.17	348	1.95
Gliridae				
*Glis glis* [Ggl]	0	0.00	1	0.01
*Muscardinus avellanarius* [Mav]	75	0.37	1	0.01
Other prey				
Mammals	6	0.03	4	0.02
Birds	174	0.85	125	0.70
Frogs	7	0.04	9	0.05
Insects	4	0.02	3	0.02
Total	20,437		17,839	

In the first scenario of data analysis, a cumulative abundance data matrix of small mammal prey compositions of the two areas was used to assess the regional differences in small mammal community structure. Within this context, we first analyzed the species richness as well as Shannon and Simpson diversity indices of consumed small mammal assemblages using individual‐based rarefaction analysis. Thereafter, we evaluated the rank‐abundance curve and composition difference of small mammal assemblages of the two areas. To account for differences in sample size between the two assemblages, rarefaction and extrapolation analyses were performed using the R package “iNEXT” (v. 3.0.1; Hsieh et al. 2024). Diversity estimates were calculated from pooled MNI data, and sampling completeness was assessed using sample coverage. Rarefaction and extrapolation curves of order *q* = 0 (species richness), *q* = 1 (Shannon diversity), and *q* = 2 (Simpson diversity) were applied. Extrapolation was performed up to 25,000 individuals, exceeding the observed abundance of the larger assemblage only slightly, and we used 1000 replicate bootstrapping runs to estimate 95% confidence intervals (Chao et al. 2014). The rarefaction curves were plotted with the package “graphics” (v. 3.6.2; R Core Team 2024). Because sample coverage approached 1.0 in both assemblages (DFP: SC = 1.000, GYB: SC = 0.999), the observed diversity estimates were considered representative of the underlying small mammal communities. To assess the difference in small mammal assemblages between the areas, non‐parametric permutational multivariate analysis of variance (PERMANOVA) with the Bray–Curtis dissimilarity index was performed from the relative MNI values with the adonis2() function in the package “vegan” (v. 2.6‐8; Oksanen et al. 2024). The FDR *p*‐value adjustment method (Benjamini and Hochberg 1995) as a post hoc test of PERMANOVA was carried out to confirm the statistically significant difference in the small mammal composition between the two areas. Principal coordinate analysis (PCoA) with betadisper() and permutes() functions in the package “vegan” was applied to visualize the results of PERMANOVA.

Rank‐abundance curves (RACs) were generated from the species‐specific cumulative MNI values pooled for each study area using the package “goeveg” (v. 0.7.5; von Lampe and Schellenberg 2024). The curves display species ranked by their relative abundance on a logarithmic scale, allowing comparison of community dominance structure between the two investigated areas. To compare RACs of the two different assemblages, the “codyn” package (v. 2.0.5; Hallett et al. 2020) with the RAC_difference() function was used (Avolio et al. 2019). Four RAC difference parameters were calculated: species richness difference (S.D.), evenness difference (E.D.), rank difference (R.D.), and species difference (Sp.D.). Species richness difference (S.D.) represents the difference in RAC length between assemblages and ranges from −1 to 1, with larger absolute values indicating greater differences in species richness. Evenness difference (E.D.) quantifies differences in assemblage evenness and also ranges from −1 to 1, where negative values indicate lower evenness and positive values indicate higher evenness in the second assemblage relative to the first. Rank difference (R.D.) measures changes in species rank abundance and ranges from 0 to 0.5, with 0.5 indicating the maximum possible rank shift between assemblages. Species difference (Sp.D.) quantifies species turnover and reflects differences in community composition resulting from species replacement. The sum of S.D. and Sp.D. corresponds to the Jaccard dissimilarity index (β‐diversity) as described by Avolio et al. (2019) and Carvalho et al. (2012).

In the second scenario of data analysis, we assessed the temporal trend of the small mammal prey diversity. First, we calculated the species richness, Shannon diversity, and Simpson dominance indices from the data of each nesting pair (breeding locality) in the two areas. Based on the resulting hierarchical data structure, we examined the spatial and temporal variations in community parameters using generalized additive mixed models (GAMMs) with the gam() function and “mgcv” package (Wood 2011). GAM modeling is suitable to detect non‐linear relationships between the variables, which are often present in ecological data (Hastie and Tibshirani 1990; Maloney et al. 2012; Wood 2017). Species richness, Shannon diversity, and Simpson dominance were analyzed as separate response variables, with year included as a smooth term and nesting pair localities included as a random effect to account for repeated observations from the same localities. Area was included as a fixed effect, and region‐specific temporal trends were modeled where supported by the data. When building the models, the error distribution and link function were selected according to the statistical properties and scale of each response variable. Species richness (S), being count data with potential overdispersion, was modeled using a negative binomial distribution with a log link. Shannon diversity (H) was modeled using a Gaussian distribution with an identity link when model diagnostics supported the corresponding assumptions. Simpson dominance (D), expressed as a continuous proportion bounded between 0 and 1, was modeled using a beta distribution with a logit link. In this scenario, four candidate GAM regression models and smoother terms were derived using penalized regression splines of the independent variables (year and area). The first model included the interaction of two predictor variables (M1: response variable (rv) ~ s(Year, by = Area, k = 8) + s(Loc, bs = “re”)), using the “by” function, which parameterizes a linear interaction to a smoothed term (Wood 2017). In addition, we tested together the main effect of year as a smoothed term and area as a parametric term (M2: rv ~ s(Year, k = 8) + Area + s(Loc, bs = “re”)), while we also tested the previous two effects separately in two additional models (M3: rv ~ s(Year, k = 8) + s(Loc, bs = “re”), M4: rv ~ Area + s(Loc, bs = “re”)).

In the third scenario, we analyzed the temporal dynamics of prey community structure. Rank change, rank shift, and turnover of the small mammal prey community were calculated with the RAC_change() function in the “codyn” package between consecutive years to analyze the consumption of Western Barn Owls between the two areas (Avolio et al. 2019; Hallett et al. 2016). We modeled these community‐level indicators using generalized additive models (GAMs) and did not include a random effect in the models since these parameters were calculated at the annual community level rather than based on repeated observations of individual nesting sites. The response distribution and link function were also selected according to the statistical properties and range of each community dynamic index. Turnover, expressed as a proportion bounded between 0 and 1, was modeled using a beta distribution with a logit link. Rank change was modeled using a Gamma distribution with a log link, whereas rank shift was modeled using a Gaussian distribution with an identity link. In this case as well, the four candidate models were the same ones described in detail above (M1–M4), with *k* = 10 and no random effect.

In the fourth scenario, we analyzed species‐specific temporal responses and the impact of environmental drivers on the abundance of prey taxa in the two different community compositions with the “mvabund” statistical framework. This procedure provides model‐based methods for analyzing multivariate abundance data and addresses several limitations associated with distance‐based community analyses (Warton et al. 2012). Species‐specific minimum number of individuals (MNI) counts were modeled using the manyglm() function of the “mvabund” package (Wang et al. 2020). This approach fits a separate generalized linear model (GLM) for each prey taxon while assessing the multivariate response at the community level through model‐based resampling procedures. To account for differences in total prey abundance among pellet samples, the logarithm of the total MNI recorded in each sample was included as an offset term. Consequently, the analysis evaluated variation in relative prey community composition rather than absolute prey abundance.

Three alternative model formulations were used to examine different potential sources of variation in prey community composition. Based on Area and Year effects, three models were built, which assessed spatial and temporal variation and included sampling area (Area) and year (Year) as main effects and their interaction (Area × Year) as explanatory variables. Of the three models, the best‐fitting model was selected by LR test and used as the first formulation. For the weather parameters, one model evaluated the effects of absolute weather conditions and included temperature (T) and rainfall (R) as explanatory variables, and another model assessed the effects of deviations from reference weather conditions (mean value of the 24‐year dataset) and included temperature and rainfall anomalies (DevT and DevR). Absolute weather variables and their corresponding anomalies were analyzed in separate models because these alternative representations of environmental variation may be correlated. Separating them into alternative model formulations therefore avoided their simultaneous inclusion in the same model and reduced the potential for multicollinearity.

Because the response variables consisted of overdispersed count data, all models were fitted using a negative binomial error distribution with a shrinkage estimator for the residual correlation structure (cor.type = “shrink”). Predictor significance was assessed using multivariate Wald‐type tests with PIT‐trap resampling, implemented using the anova.manyglm() function. The three model formulations were interpreted as complementary analyses addressing, respectively, spatial and temporal variation in community composition, the effects of absolute weather conditions, and the effects of anomalous weather conditions.

Based on the mvabund framework and the manyglm() method, the small mammal prey taxa were selected by the significant Wald test. After that, the temporal dynamics of the abundance of these selected prey taxa were also analyzed by GAMMs. In the case of the taxon‐specific change of abundance, the same four candidate models were run as in the analysis of changes in community parameters over time with *k* = 5 in the smooth term, and to account for differences in total prey abundance among samples, the logarithm of the total prey abundance (log_total) was included as an offset term in the GAMMs.

For each scenario in which GAMs or GAMMs were used, model selection was based on the deviance explained (%) and adjusted *R* ^2^ values, where applicable, together with the REML criterion (Wood 2017). The best‐fitting models were selected from the candidate models based on restricted maximum likelihood (REML) scores, with lower REML values generally indicating a better fit among comparable models (Wood 2011). The statistical significance of predictor variables and their interactions was assessed using Type III analysis of variance (ANOVA) tests implemented with the anova.gam() function in R. Predictors or interactions were considered statistically significant when *p* < 0.05.

To further characterize the relationships between the response and predictor variables, marginal effects were estimated using the “modelbased” package (Makowski et al. 2025). For selected models, estimated slopes were calculated to quantify the local rate of change in the predicted response along the predictor variable. Where applicable, Johnson–Neyman intervals were used to identify regions of the predictor space in which the estimated slope differed significantly from zero. These significant intervals were indicated in the corresponding summed‐effect plots. Additionally, we performed marginal contrast analysis by estimating the differences between each level of a nominal factor, using the estimate_contrasts() function in the “modelbased” package. The partial effects of the selected smooth terms were visualized using the plot() function from the graphics package (version 3.6.2; R Core Team 2024). In the case of the parametric term, the estimated parameters were visualized by barplots (“graphic” package), and the parametric effect plot was generated by the plot_parametric() function in the “itsadug” package (v. 2.4.1; van Rij et al. 2022). Based on the applied nominal predictor (area), a potential difference between smooth terms of categorical predictor subsets was tested using the plot_diff() function in the “itsadug” R package (v. 2.4.1; van Rij et al. 2022). Summed‐effect plots were generated using partial conditional plots on the response scale with the “visreg” package (Breheny and Burchett 2017). Marginal effects and estimated slopes were visualized using the estimated_slope() function from the “modelbased” package.

## 3 Results

During the 24‐year monitoring of the Western Barn Owl's food habits in the two areas, a total of 39 animal taxa and 38,276 prey items were identified from the examined pellet samples (Table 1). Small mammals were the most frequent in the barn owl's diet in both investigated areas (DFP: 99.111% ± 0.190%; GYB: 99.217% ± 0.142%). The proportion distribution of common voles significantly differed between the two landscapes (*χ* ^2^ = 288.89, *p* < 0.001); the consumption of this main prey of barn owls was larger in the DFP. The relative abundance of *Apodemus* wood mice as an alternative prey group (*Apodemus* spp.) (*χ* ^2^ = 1289.50, *p* < 0.001) and the striped field mouse (*χ* ^2^ = 461.89, *p* < 0.001) was significantly higher in the DFP than in the northwestern area (GYB). In contrast, the percentage distribution of the common shrew abundance was significantly higher in the GYB (*χ* ^2^ = 3791.20, *p* < 0.001), which reflected that this species is the typical alternative prey for the Western Barn Owls. Regarding the shrew consumption of owls, we also tested the proportion distribution of the Crocidura genus, which significantly differed in the comparison of the two investigated areas (*χ* ^2^ = 106.73, *p* < 0.001). The relative abundance of this prey group was higher in the DFP, which indicates that these species are important alternative prey in this region (Table 1).

### 3.1 Regional Differences in Small Mammal Community Structure

**FIGURE 2:**
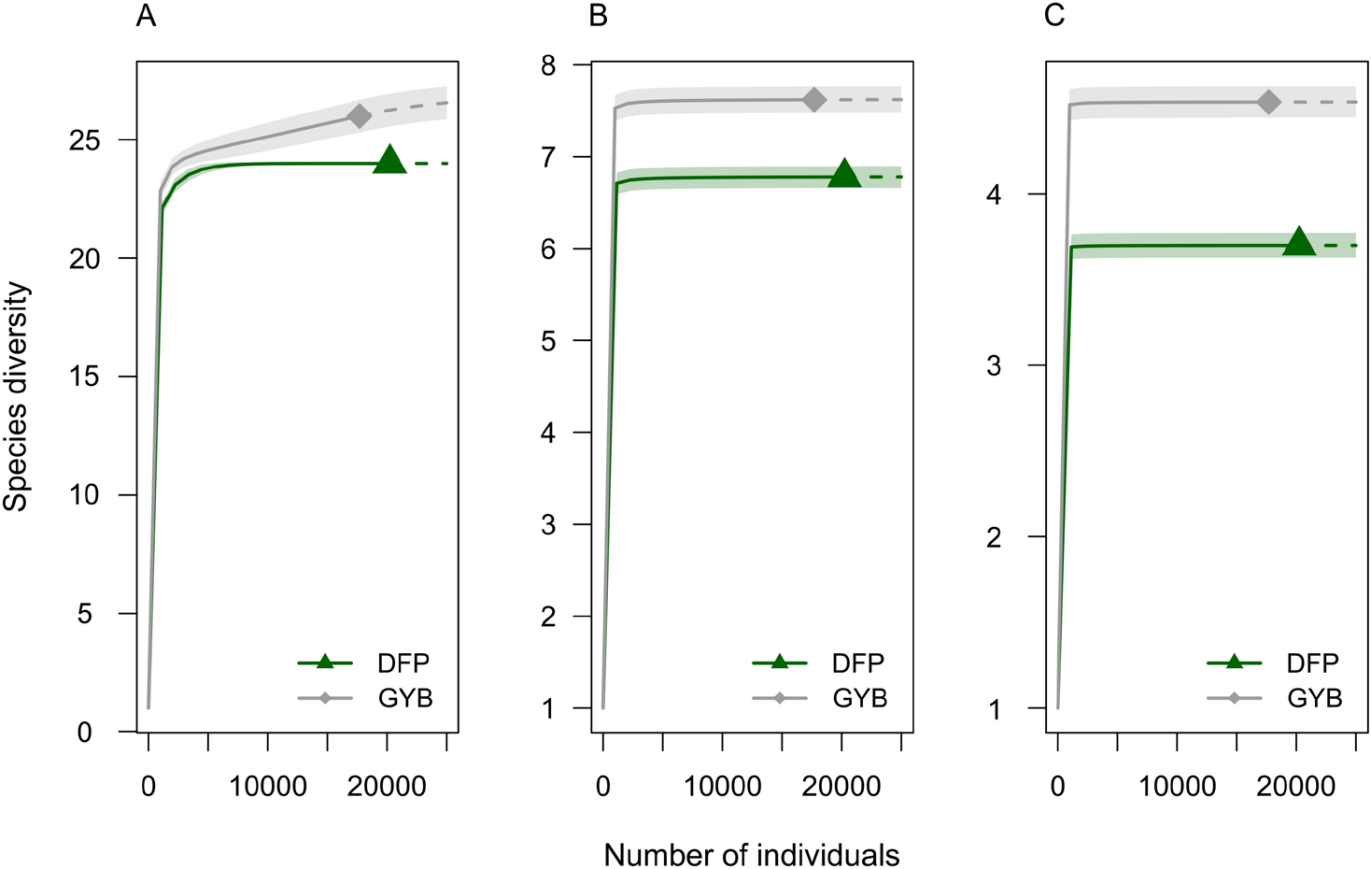
Rarefaction curves illustrating the species richness (*q* = 0) (A), Shannon diversity (*q* = 1) (B) and Simpson diversity (*q* = 2) (C) of the small mammal assemblages of the two investigated areas.

Based on the asymptotic diversity estimates calculated via iNEXT in the first scenario, the GYB community exhibited consistently and statistically significantly higher biodiversity than the DFP community across all three investigated orders of Hill numbers (*q* = 0, 1, 2). This significance is statistically supported by the non‐overlapping 95% confidence intervals (CI) between the two sites. Both communities were represented by large sample sizes, providing a robust basis for the diversity comparisons. The iNEXT curves approached an asymptote for both sites, indicating that sampling captured most of the detectable diversity. The high sample coverage values further support this interpretation. Although the estimates were based on interpolation and extrapolation to account for differences in sample completeness and facilitate comparison, extrapolation resulted in little to no change in the estimated qD values. This close agreement between observed/interpolated and extrapolated diversity estimates indicates that the sampling effort was sufficient and that the resulting diversity patterns are robust. The estimated asymptotic species richness was higher for the GYB community (^0^ *D̂* = 27.00; 95% CI: 26.38–27.62) than for the DFP community (^0^ *D̂* = 24.00; 95% CI: 24.00–24.00). For DFP, the observed and estimated richness were identical with a standard error of 0, indicating that the species pool was fully sampled at this site. The GYB community showed a significantly higher Shannon diversity (^1^ *D̂* = 7.63; 95% CI: 7.46–7.79) compared to the DFP community (^1^ *D̂* = 6.78; 95% CI: 6.64–6.92). Simpson diversity (*q* = 2) was significantly higher in the GYB community, with an estimated diversity of ^2^ *D̂* = 4.54 (95% CI: 4.43–4.64), compared to ^2^ *D̂* = 3.70 (95% CI: 3.62–3.78) in the DFP community, indicating greater diversity among dominant species in GYB. The consistent separation of the diversity estimates across *q* = 0, 1, and 2, combined with the asymptotic behavior of the curves, high sample coverage, and the minimal effect of extrapolation (^1^ *D̂*: DFP_Δ(extrapol‐obs)_ = 0.000811, GYB_Δ(extrapol‐obs)_ = 0.001968; ^2^ *D̂*: DFP_Δ(extrapol‐obs)_ = 0.000094, GYB_Δ(extrapol‐obs)_ = 0.000264), indicates that the observed differences between the two communities are unlikely to be artifacts of insufficient sampling (Figure 2).

**FIGURE 3:**
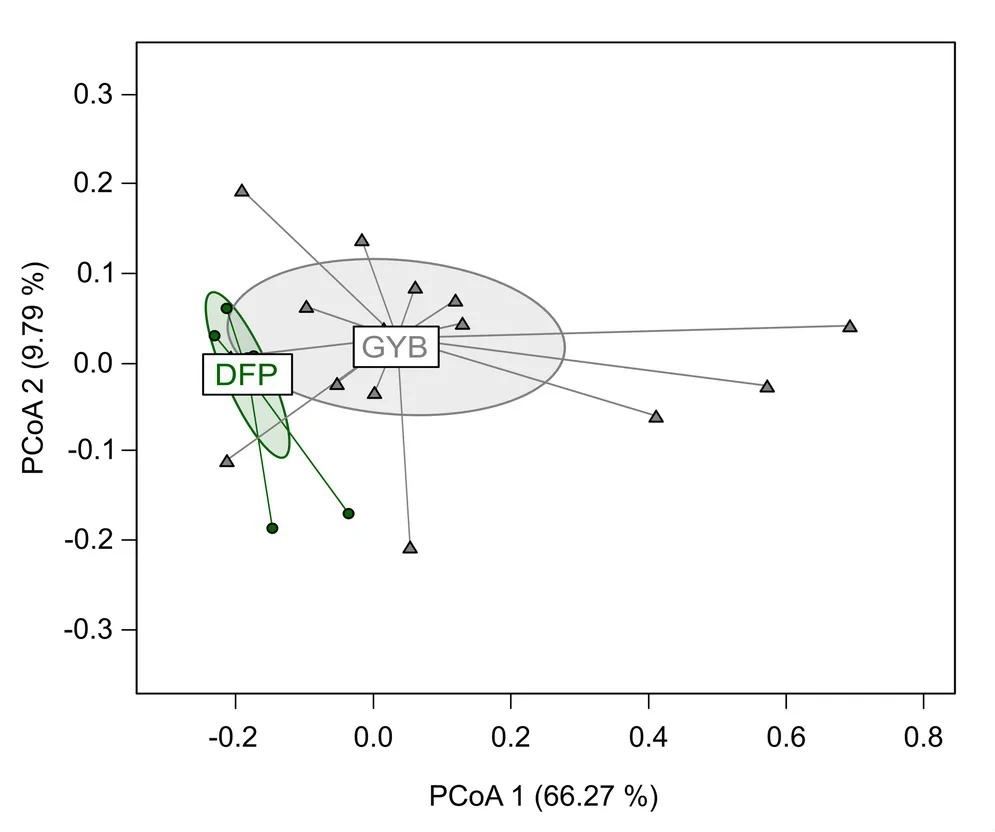
Principal coordinates analysis (PCoA) based on Bray–Curtis dissimilarity matrices of small mammal assemblages in the DFP (green circles) and in the GYB (gray triangles) (ellipses indicate 95% confident intervals).

According to the result of the non‐parametric PERMANOVA, the diet composition of Western Barn Owls significantly differed between the two investigated areas (*F* = 6.042, *R* ^2^ = 0.195, *p* = 0.006). Pairwise comparisons confirmed a significant difference between the two areas (DFP vs. GYB: FDR‐*p* = 0.0039). The first two axes of the PCoA ordination account for 76.06% of the total variance; the majority of this variation was carried by the PCoA1 axis (66.27%) (Figure 3). The homogeneity of multivariate dispersion showed a marginal, non‐significant trend (betadisper: *F* = 3.974, *p* = 0.057). Therefore, the significant PERMANOVA result is supporting the interpretation of genuine differences in community composition between the two areas.

**FIGURE 4:**
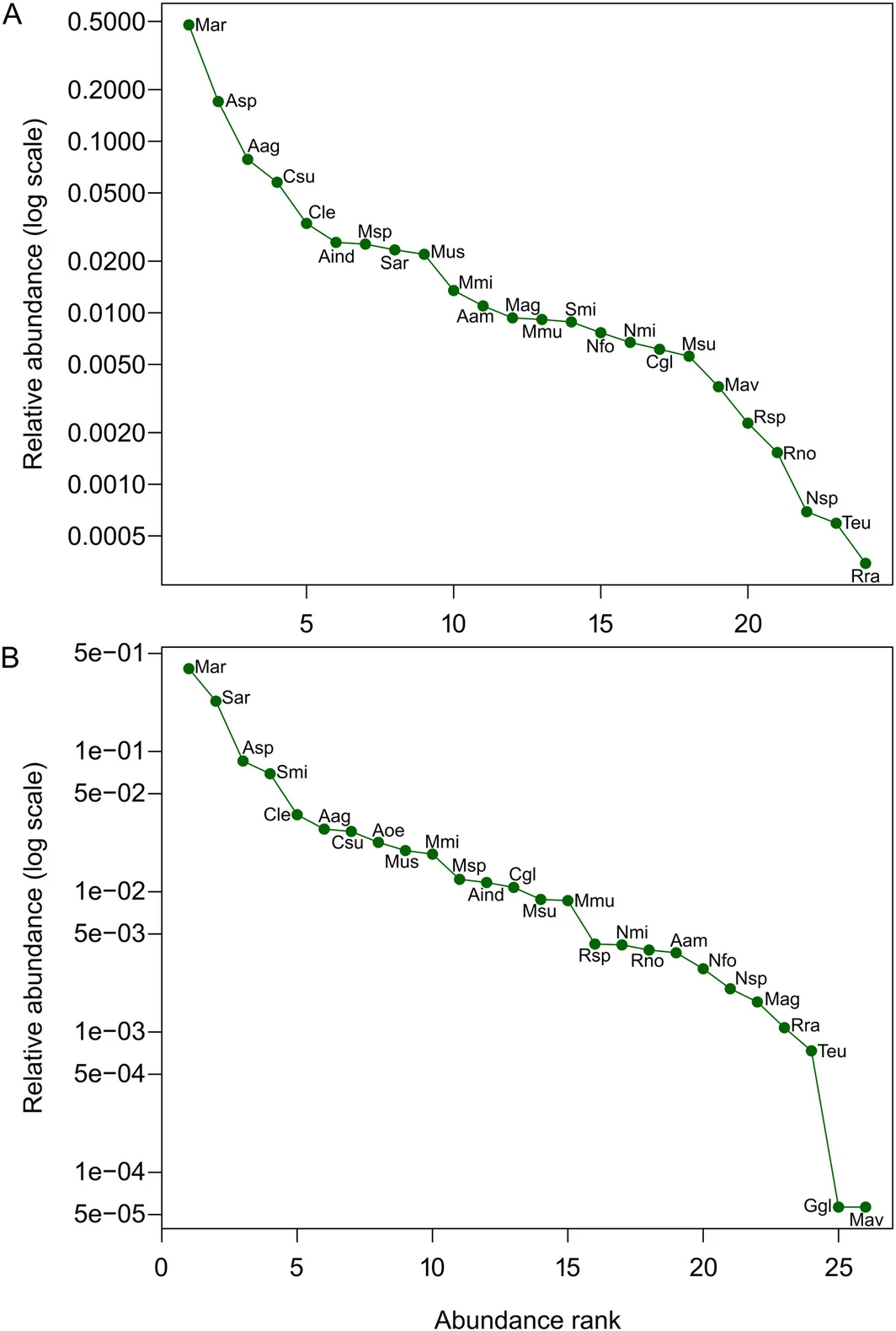
Rank abundance curves of the small mammal assemblages in the DFP (A) and in the GYB (B).

Based on the regional difference of dominance structure, the rank‐abundance distribution of Western Barn Owls' prey composition was different between the two investigated lowland regions, as demonstrated by the shape of the rank‐abundance curves (Figure 4A). Although the length of the curves was very similar, the species richness of prey was higher in the GYB. In both areas, the common vole was the most frequent prey. In the case of the DFP, *Apodemus* spp. was the second, and the striped field mouse was the third most frequent prey (Figure 4B). In contrast, the common shrew was the second most preyed species, and the third was the *Apodemus* spp. in the GYB.

Comparative RAC analysis revealed only small differences between the two small mammal assemblages represented in the Western Barn Owl diet. Species richness difference was low (S.D. = 0.077), indicating slightly higher species richness in the northern area (GYB) than in the southern area (DFP). The evenness difference was also small (E.D. = −0.066), indicating highly similar community structures. Rank differences were low (R.D. = 0.149), corresponding to 29.8% of the maximum possible rank change, suggesting only minor shifts in species dominance. No species turnover was detected (Sp.D. = 0). Consequently, the combined value of S.D. and Sp.D., corresponding to Jaccard dissimilarity (β‐diversity), indicated very low beta diversity between the two small mammal assemblages.

### 3.2 Temporal Trends in Small Mammal Prey Diversity

**FIGURE 5:**
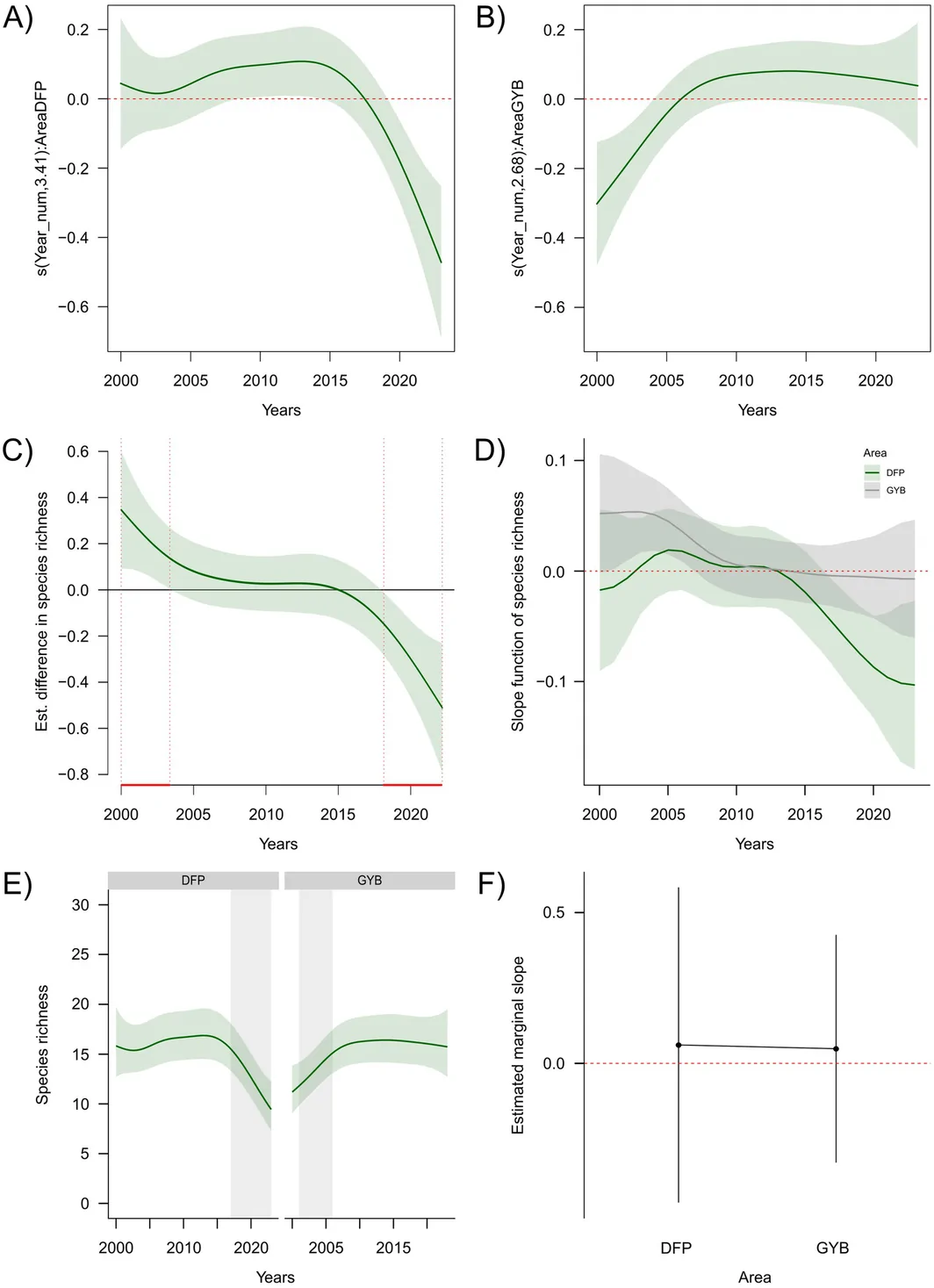
GAMM plots showing the effect of the interaction of years and the two investigated areas on the values of the species richness (S). (A) Smooth spline of years as a continuous explanatory variable in the case of the DFP. (B) Smooth spline of years as a continuous explanatory variable in the case of the GYB. (C) The smooth difference plot of the spline functions between two areas, the red lines indicate the periods when the species richness significantly differed between the two investigated areas. (D) Estimated slopes, where the red dashed line denotes a slope of zero (no change). (E) Nonlinear summed effect of years, where the vertical gray bars mark significant marginal effect (slope), showing the intervals in which the rate of change in the response variable was statistically confirmed. (F) Comparison of the slopes between the DFP and the GYB, where the red dashed line denotes a slope of zero.

In the second scenario, the best GAMM model (S ~ s(Year_num, by = Area) + s(Loc, bs = “re”); deviance explained 34.2%; *R* ^2^_adj_ = 0.294, REML = 685.940) revealed that the interaction of years and areas had a major impact on the species richness. The smooth spline of the response variable showed a non‐linear effect in both areas, but the shapes of the function were different. In the case of the DFP, the smooth term was significant (edf = 3.412, *χ* ^2^ = 20.400, *p* < 0.001). The visualization of the smooth term for DFP confirms a significant temporal trend; the spline reached a significant positive peak around 2014, followed by a decreasing trend (Figure 5A). The smooth term was also significant in the case of the GYB (edf = 2.678, *χ* ^2^ = 13.130, *p* = 0.007). Following an initial increase in the first third of the period, species richness remained largely unchanged (Figure 5B). Based on the plot difference function, there were significant differences in the species richness between 2000–2004 and 2018–2023 (Figure 5C). The random effect for locations was also significant (edf = 15.442, *χ* ^2^ = 54.410, *p* < 0.001). The marginal effect analysis showed a significant positive slope between 2017 and 2023 (slope: 0.15–0.42, *z* = 2.13–6.68, *p* < 0.05) and a significant negative slope in the decreasing trend between 2015 and 2019 (slope: (−0.10)–(−0.05), *z* = 2.40–3.69, *p* < 0.05) in DFP, while this analysis showed a significant positive slope in the increasing trend of the function between 2001 and 2006 (slope: 0.04–0.05, *z* = 2.02–3.07, *p* < 0.05) in GYB (Figure 5D). The function intervals where the estimated slope was significant were marked on the partial conditional plot generated on the response scale in both areas (Figure 5E). In the analysis of the nominal variable, the estimated slopes of both areas were positive but not significant (DFP: slope = 0.06 ± 0.27 SE, *z* = 0.23, *p* = 0.819; GYB: slope = 0.05 ± 0.19 SE, *z* = 0.25, *p* = 0.801) (Figure 5F). The marginal effect contrast between the two areas was not significant (GYB vs. DFP difference = −0.37 ± 0.86 SE, *z* = −0.44, *p* = 0.664).

**FIGURE 6:**
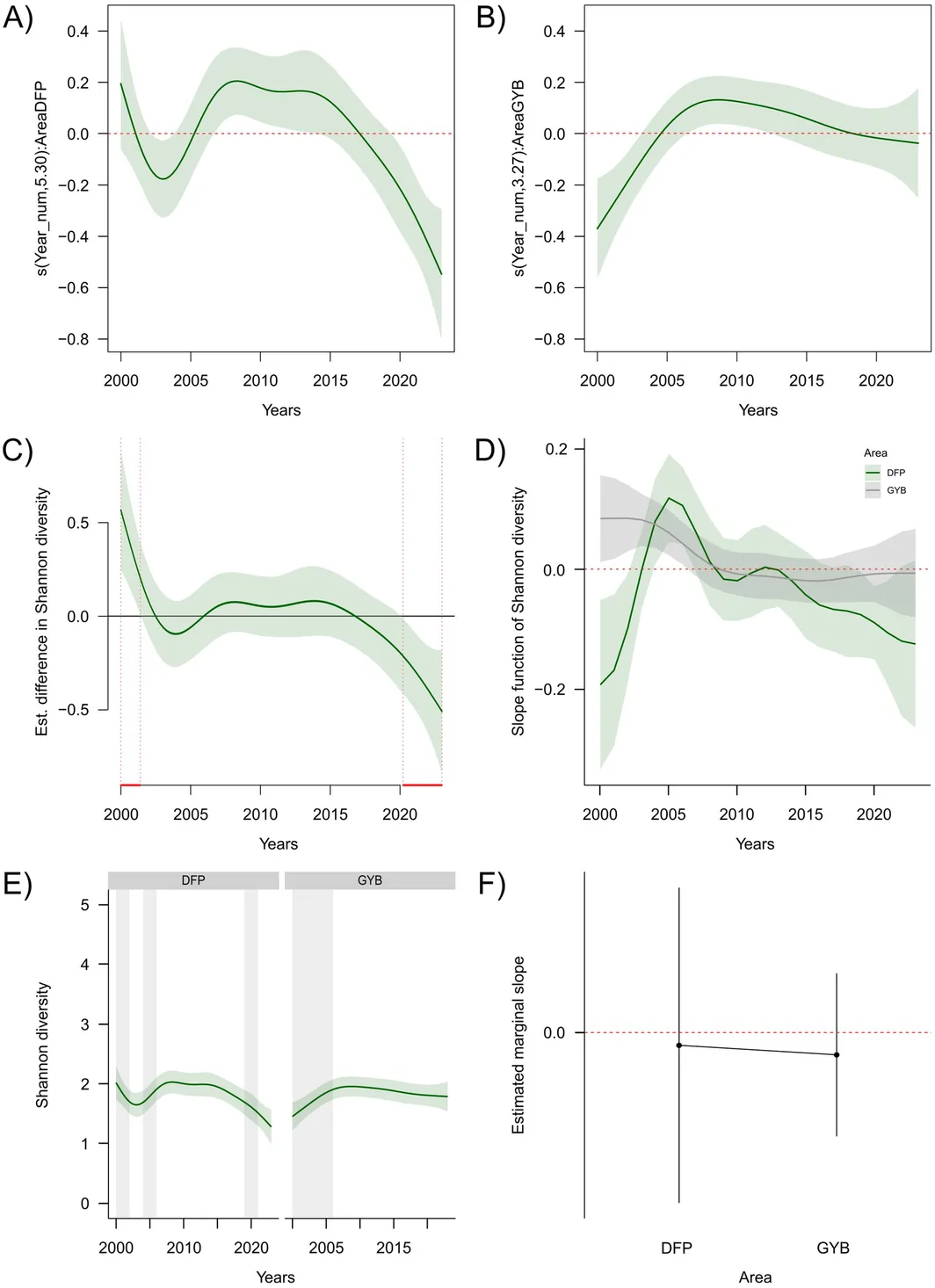
GAMM plots showing the effect of the interaction of years and the two investigated areas on the values of the Shannon diversity (H). (A) Smooth spline of years as a continuous explanatory variable in the case of the DFP. (B) Smooth spline of years as a continuous explanatory variable in the case of the GYB. (C) The smooth difference plot of the spline functions between two areas, the red lines indicate the periods when the Shannon diversity significantly differed between the two investigated areas. (D) Estimated slopes, where the red dashed line denotes a slope of zero (no change). (E) Nonlinear summed effect of years, where the vertical gray bars mark significant marginal effect (slope), showing the intervals in which the rate of change in the response variable was statistically confirmed. (F) Comparison of the slopes between the DFP and the GYB, where the red dashed line denotes a slope of zero.

Regarding the Shannon diversity, the best model (H ~ s(Year_num, by = Area) + s(Loc, bs = “re”); deviance explained 42.0%; *R* ^2^_adj_ = 0.348, REML = 111.590) demonstrated that the interaction of years and areas (Area) had a significant effect on the Shannon diversity. In both areas, the smooth spline of the response variable indicated a non‐linear effect. Regarding the DFP, the smooth term was significant (edf = 5.302, *F* = 5.915, *p* < 0.001). The visualization of the smooth term for DFP confirms a significant temporal trend; the spline reached a significant negative low point in 2003, followed by an increasing trend until 2008, and then the Shannon diversity decreased (Figure 6A). The smooth term was also significant in the case of the GYB (edf = 3.269, *F* = 4.634, *p* < 0.001). The Shannon diversity increased until 2008; after that a slight decreasing trend was observed (Figure 6B). Based on the plot difference function, there were significant differences in the Shannon diversity between 2000–2001 and 2020–2023 (Figure 6C). The random effect for locations was also significant (edf = 16.598, *F* = 2.371, *p* < 0.001). The marginal effect analysis of the DFP function showed a significant negative slope between 2000 and 2002 (slope: (−0.19)–(−0.10), *t* = 2.14–2.68, *p* < 0.05) as well as between 2019 and 2021 (slope: (−0.11)–(−0.08), *t* = 2.09–3.50, *p* < 0.01) and a significant positive slope between 2004 and 2006 (slope: 0.08–0.12, *t* = 2.20–3.23, *p* < 0.01). On the other hand, the marginal effect analysis of the GYB function showed a significant positive slope between 2000 and 2006 (slope: 0.08–0.11, *t* = 2.22–3.23, *p* < 0.05) (Figure 6D). The statistically significant slope intervals were marked on the partial conditional plot in both areas (Figure 6E). In the analysis of the nominal variable, the estimated slopes of the two areas were negative but not significant (DFP: slope = −5.91e‐3 ± 0.04 SE, *t* = −0.16, *p* = 0.874; GYB: slope = −0.01 ± 0.02 SE, *t* = −0.53, *p* = 0.593) (Figure 6F). The marginal effect contrast between the two areas was not significant for this species (GYB vs. DFP difference = −0.05 ± 0.08 SE, *t* = −0.61, *p* = 0.545).

**FIGURE 7:**
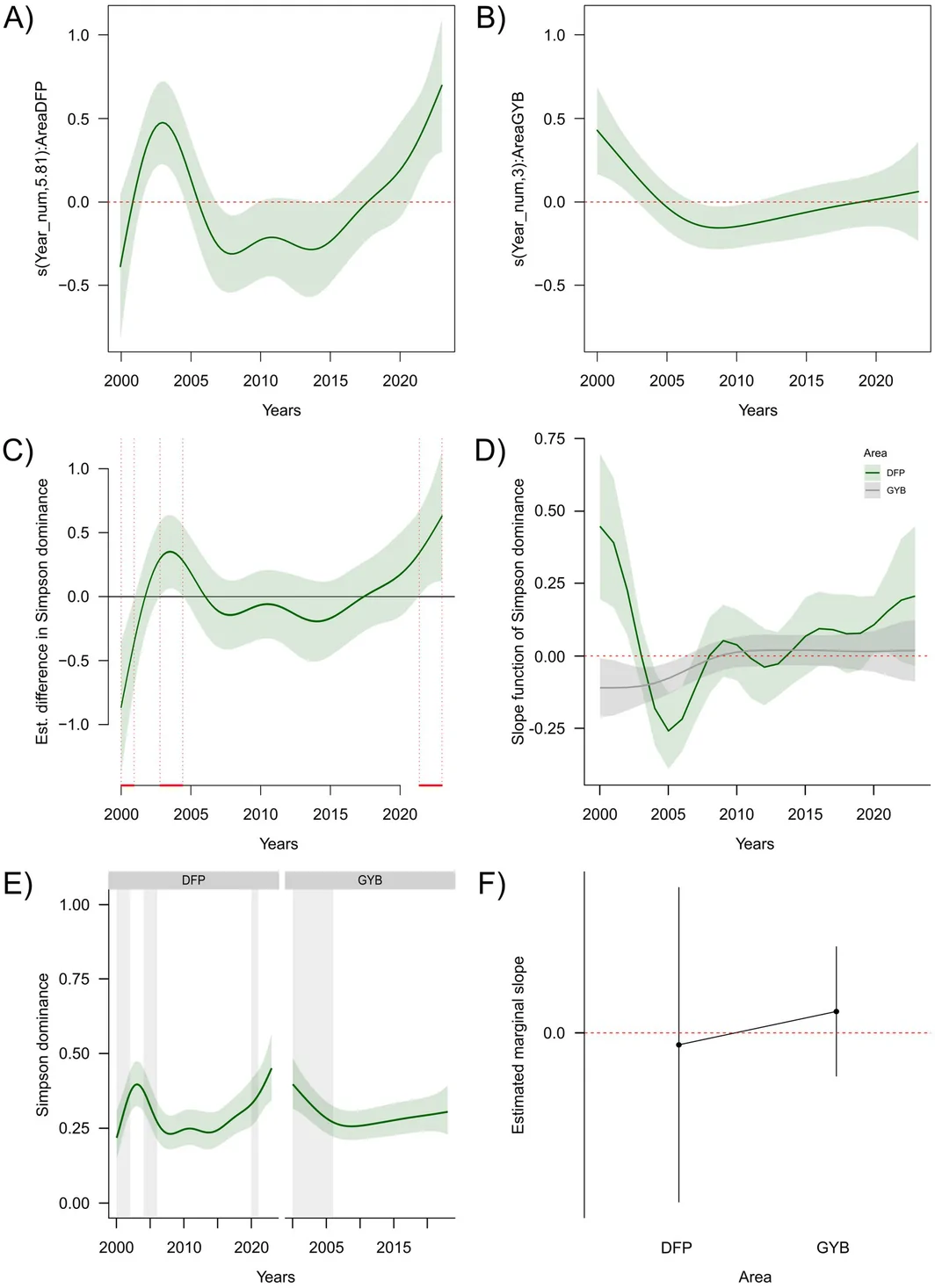
GAMM plots showing the effect of the interaction of years and the two investigated areas on the values of the Simpson dominance (D). (A) Smooth spline of years as a continuous explanatory variable in the case of the DFP. (B) Smooth spline of years as a continuous explanatory variable in the case of the GYB. (C) The smooth difference plot of the spline functions between two areas, the red lines indicate the periods when the Simpson dominance significantly differed between the two investigated areas. (D) Estimated slopes, where the red dashed line denotes a slope of zero (no change). (E) Nonlinear summed effect of years, where the vertical gray bars mark significant marginal effect (slope), showing the intervals in which the rate of change in the response variable was statistically confirmed. (F) Comparison of the slopes between the DFP and the GYB, where the red dashed line denotes a slope of zero.

In the case of Simpson dominance, the best candidate model (D ~ s(Year_num, by = Area) + s(Loc, bs = “re”); deviance explained 37.3%; *R* ^2^_adj_ = 0.301, REML = −161.310) demonstrated that the interaction of years and areas (Area) significantly influenced this parameter. In both areas, the smooth spline of the response variable indicated a non‐linear effect; however, the shapes of the function were different. Regarding DFP, the smooth term was significant (edf = 5.807, *χ* ^2^ = 32.130, *p* < 0.001). The visualization of the smooth term for DFP confirmed a significant temporal trend, the spline reaching a significant positive peak around 2013, followed by a decreasing trend, then after a period of stagnation, dominance increased again from 2015 (Figure 7A). The smooth term was also significant in the case of the GYB (edf = 2.996, *χ* ^2^ = 33.510, *p* = 0.007). The Simpson dominance decreased until 2009; after that, a slight increasing trend was observed (Figure 7B). Based on the plot difference function, there were significant differences in the Simpson dominance between 2000–2001, 2002–2005, and 2021–2023 (Figure 7C). The random effect for locations was also significant (edf = 14.438, *χ* ^2^ = 43.510, *p* < 0.001). The marginal effect analysis showed a significant positive slope between 2000 and 2002 (slope: 0.23–0.45, *z* = 2.87–3.50, *p* < 0.01) and a significant negative slope between 2004 and 2006 (slope: (−0.26)–(−0.18), *z* = 2.77–3.88, *p* < 0.01) in DFP. In contrast, the estimated marginal effect in GYB was significantly negative in the decreasing trend of the function between 2000 and 2006 (slope: (−0.11)–(−0.06), *z* = 2.12–3.21, *p* < 0.05) (Figure 7D). The statistically significant marginal effects were marked on the partial conditional plot in both areas (Figure 7E). In the case of the nominal variable (Area), the estimated slope of DFP was negative, while that of the GYB was positive, and none of them were significant (DFP: slope = −1.86e‐3 ± 0.01 SE, *z* = −0.15, *p* = 0.882; GYB: slope = 3.35e‐3 ± 5.18e‐3 SE, *z* = 0.65, *p* = 0.518) (Figure 7F). The marginal effect contrast between the two areas was not significant (GYB vs. DFP difference = 0.01 ± 0.02 SE, *z* = 0.48, *p* = 0.628).

### 3.3 Temporal Dynamics of Prey Community Structure

**FIGURE 8:**
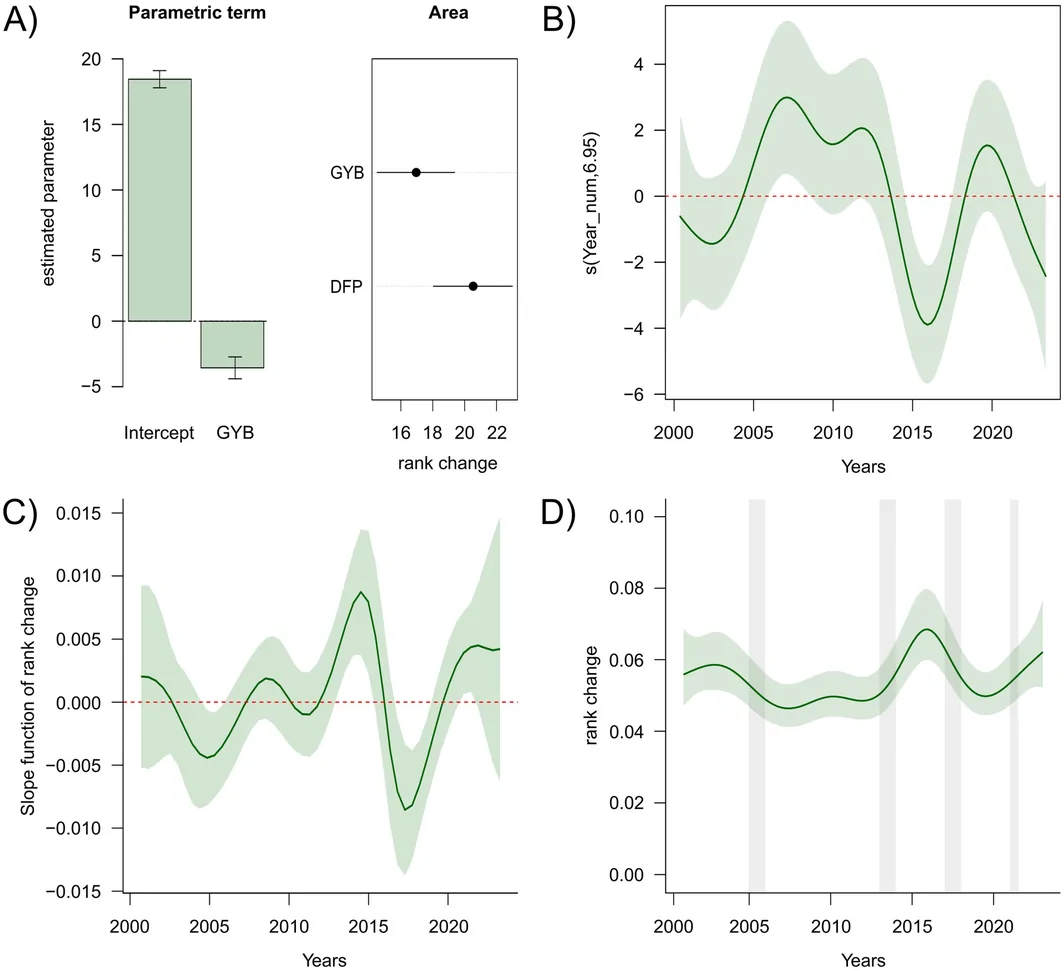
GAM plots showing the effect of the two investigated areas and years on the values of rank change. (A) Parametric term effect plots with the estimated coefficients of areas are shown on the left side, and their fitted effects are shown on the right side. (B) Smooth spline of years as a continuous explanatory variable. (C) Estimated slope, where the red dashed line denotes a slope of zero (no change). (D) Nonlinear summed effect of years, where the vertical gray bars mark significant marginal effect (slope), showing the intervals in which the rate of change in the response variable was statistically confirmed.

In the third scenario, temporal changes of community parameters were assessed by GAM. Regarding the rank change, the best GAM model (rank change ~ s(Year) + Area; deviance explained 60.0%; *R* ^2^_adj_ = 0.525, REML = −132.850) revealed that the years and areas (Area) had a major impact on this community dynamic index. The parametric term of this candidate model showed a significant effect of area (*F* = 18.200, *p* = 0.005), which demonstrated that rank change in the DFP was significantly higher than in the GYB (estimated parameter = −3.554 ± 0.833 SE, *t* = 4.266, *p* < 0.001) (Figure 8A). Based on the results of the smooth term, the year had a significant effect on the temporal change of this community dynamic characteristic. The GAMM smooth spline showed a non‐linear fluctuating effect of years (edf = 6.947, *F* = 3.506, *p* = 0.005) (Figure 8B). The marginal effect analysis of the year effect showed two significant negative and two significant positive slope intervals (2005–2006: slope: 1.46–1.55, *z* = 2.01–2.38, *p* < 0.05; 2013–2014: slope: (−2.44)–(−2.04), *z* = 3.34–3.62, *p* < 0.001; slope: 2.05–2.30, *z* = 3.45–3.54, *p* < 0.01; slope: −1.46, *z* = 2.48, *p* = 0.013) (Figure 8C). The statistically significant slope intervals where rank change showed significant change were marked on the partial conditional plot with the response scale (Figure 8D). The marginal effect contrast between the two areas was significant (GYB vs. DFP difference = 0.01, *t* = 3.74. *p* < 0.001). The marginal effect contrast between the two areas was significant (GYB vs. DFP difference = 0.01 ± 2.73e‐03 SE, *t* = 3.74, *p* < 0.001).

In the case of the rank shift, the best‐fitting candidate model included the geographic area as a parametric term only (rank shift ~ Area), which model explained 7.31% of the total deviance (*R* ^2^_adj_ = 0.051, REML = −45.028). Based on the results of the parametric term, the importance of area in this model was not significant (*F* = 3.31, *p* = 0.076), and this community dynamic characteristic did not differ in the comparison of the two areas (estimated parameter = 0.042 ± 0.023 SE, *t* = 1.819, *p* = 0.076). In respect of turnover, the best model was also the one that took only area into account (turnover ~ Area), which explained 4.53% of the total deviance (*R* ^2^_adj_ = 0.012, REML = −71.564). Similarly to rank shift, the main effect of the area was not significant (*χ* ^2^ = 1.943, *p* = 0.163). Based on the results of the parametric term, no significant difference in turnover was observed between the two areas (estimated parameter = 0.234 ± 0.168 SE, *z* = 1.394, *p* = 0.163).

### 3.4 Species‐Specific Temporal Responses and Environmental Drivers

Based on the “mvabund” statistical framework, the model including the interaction between Year and Area was the final model among the alternative manyglm() formulations, selected by LR tests as the best‐supported model for explaining variation in prey community composition. The significant multivariate Wald test indicated that prey composition differed between the two study areas (*χ* ^2^ = 24.955, *p* < 0.001) and over consecutive years (*χ* ^2^ = 10.774, *p* < 0.001), as well as in the case of the Area × Year interaction term (*χ* ^2^ = 7.886, *p* < 0.01).

Taxon‐specific Wald tests identified nine prey taxa showing significant Year × Area interactions: *Sorex araneus* (Sar; *χ* ^2^ = 13.461, *p* < 0.001), *Sorex minutus* (Smi; *χ* ^2^ = 8.574, *p* < 0.001), *Crocidura suaveolens* (Csu; *χ* ^2^ = 7.816, *p* < 0.001), *Arvicola amphibius* (Aam; *χ* ^2^ = 4.862, *p* = 0.020), *Apodemus agrarius* (Aag; *χ* ^2^ = 10.705, *p* < 0.001), *Apodemus* spp. (Asp; *χ* ^2^ = 7.680, *p* < 0.001), and *Mus spicilegus* (Msp; *χ* ^2^ = 5.884, *p* = 0.002). No significant Year × Area interaction was detected for the remaining taxa (*p* > 0.05).

**FIGURE 9:**
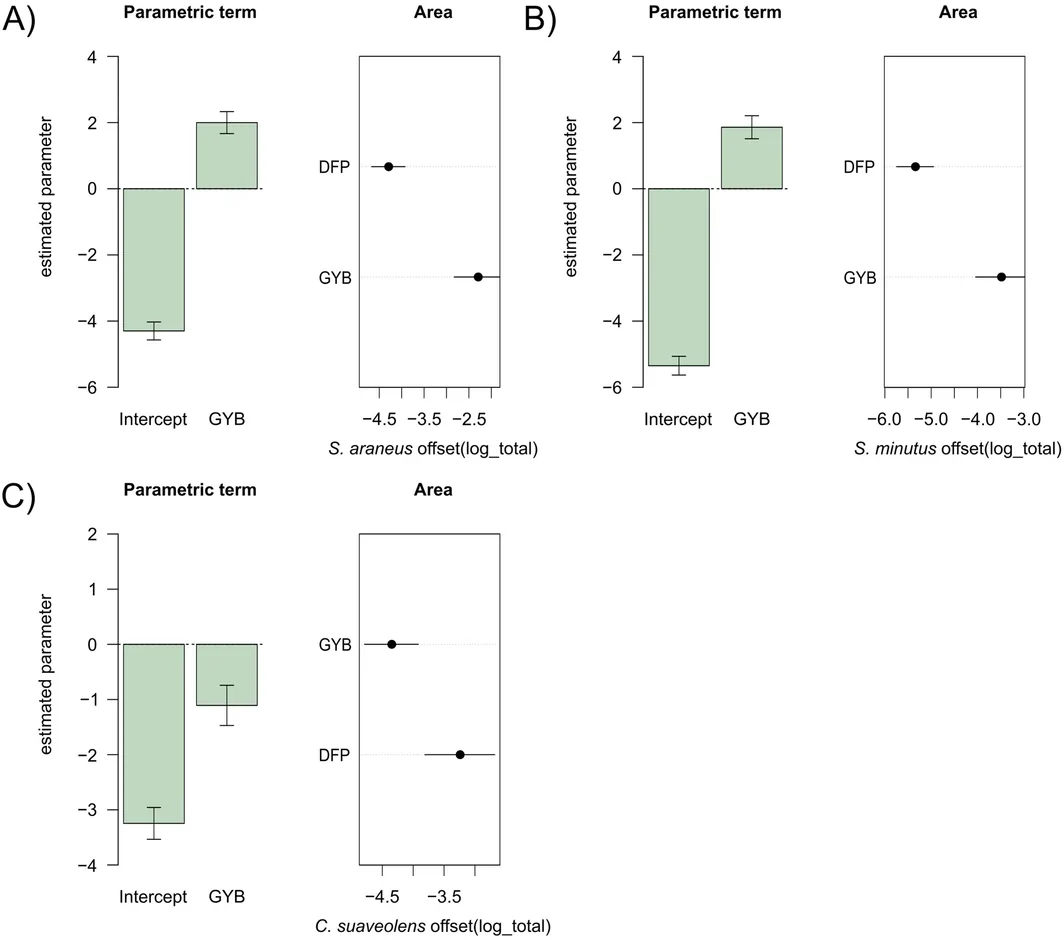
GAMM plots showing the effect of the two investigated areas on the abundance of the common shrew ( *S. araneus* ) (A), the pygmy shrew ( *S. minutus* ) (B), and the lesser white‐toothed shrew ( *C. suaveolens* ) (C). Parametric term effect plots with the estimated coefficients of areas are shown on the left side, and their fitted effects are shown on the right side.

These taxa were subsequently analyzed using generalized additive mixed models (GAMMs) to characterize their species‐specific temporal abundance dynamics and to determine how the temporal trajectories differed between the two study areas. Among the selected shrew species, the best‐fitting candidate model for the common shrew included the geographic area as a parametric term and the localities as a random effect (Sar ~ Area + s(Loc, bs = “re”) + offset(log_total)), which model explained 52.3% of the total deviance (*R* ^2^_adj_ = 0.852, REML = 657.070). The parametric term showed a significant effect of area (*χ* ^2^ = 36.230, *p* < 0.001), according to which this shrew species was more abundant in the GYB (estimated parameter = 1.998 ± 0.332 SE, *z* = 6.019, *p* < 0.001) (Figure 9A). The random effect of locations as a smooth term (edf = 17.520, *χ* ^2^ = 54.440, *p* < 0.001), as well as the marginal effect contrast between the two areas was significant (GYB vs. DFP difference = 11.27 ± 2.12 SE, *z* = 5.31, *p* < 0.001). For the pygmy shrew, the best model also includes the area as a parametric term and the localities as a random effect (Smi ~ Area + s(Loc, bs = “re”) + offset(log_total)), and this model explained 39.4% of the total deviance (*R* ^2^_adj_ = 0.843, REML = 426.050). The effect of the area was significant in this candidate model (*χ* ^2^ = 28.460, *p* < 0.001). The parametric term demonstrated that the pygmy shrew was also more abundant in the GYB (estimated parameter = 0.858 ± 0.348 SE, *z* = 5.335, *p* < 0.001) (Figure 9B). The specific sampling locations had a significant impact on abundance variation (edf = 13.460, *χ* ^2^ = 30.410, *p* < 0.001). The marginal effect contrast analysis showed a significant difference between the areas (difference = 3.26 ± 0.73 SE, *z* = 4.48, *p* < 0.001). Regarding the lesser white‐toothed shrew, the same model as for Sorex species accounted for 43.6% of the total deviance (Csu ~ Area + s(Loc, bs = “re”) + offset(log_total), *R* ^2^_adj_ = 0.771, REML = 537.010). The Area as a nominal predictor had significant importance in this model (*χ* ^2^ = 9.228, *p* = 0.002). The parametric component revealed that these drought‐tolerant shrews occurred significantly more frequently in the DFP (estimated parameter = −1.107 ± 0.364 SE, *z* = 3.038, *p* < 0.01) (Figure 9C). The random effect of locations as a smooth term was also significant for this shrew species (edf = 17.490, *χ* ^2^ = 83.640, *p* < 0.001). The marginal effect contrast between the two areas was also significant for this species (GYB vs. DFP difference = −3.59 ± 1.52 SE, *z* = −2.37, *p* = 0.018).

**FIGURE 10:**
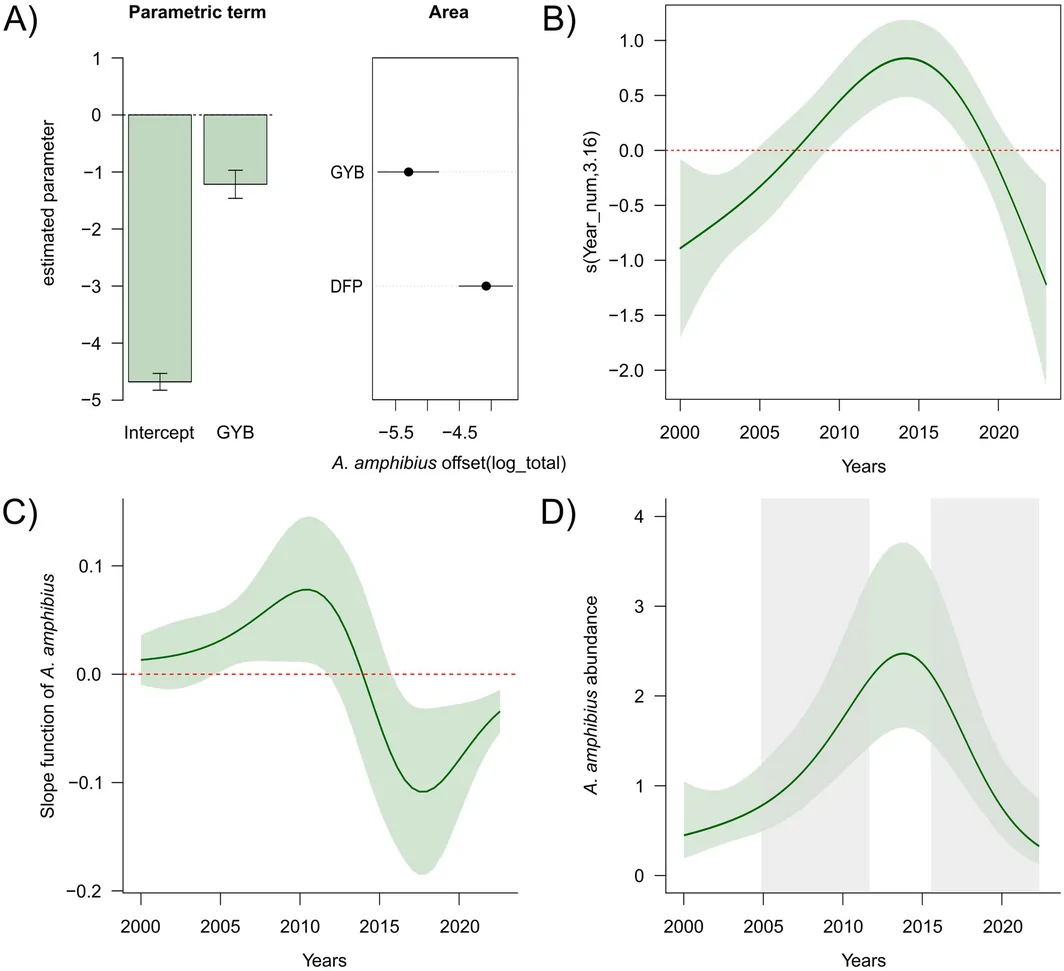
GAMM plots showing the effect of the two investigated areas and years on the abundance of the European water vole ( *A. amphibius* ). (A) Parametric term effect plots with the estimated coefficients of areas are shown on the left side, and their fitted effects are shown on the right side. (B) Smooth spline of years as a continuous explanatory variable. (C) Estimated slope, where the red dashed line denotes a slope of zero (no change). (D) Nonlinear summed effect of years, where the vertical gray bars mark significant marginal effect (slope), showing the intervals in which the rate of change in the response variable was statistically confirmed.

The best model for the European water vole included the effects of the year and geographic area as well as the random effect for specific locations (Aam ~ s(Year) + Area + s(Loc, bs = “re”) + offset(log_total)). The model explained 21.5% of the deviance (*R* ^2^_adj_ = −0.214, REML = 291.770). The Area was a significant component in this candidate model (*χ* ^2^ = 24.380, *p* < 0.001), the parametric term showed that the abundance of the water vole in the DFP was significantly higher than in the GYB (estimated parameter = −1.216 ± 0.246 SE, *z* = 4.937, *p* < 0.001) (Figure 10A). Based on the results of the smooth term, the year was a significant parameter in this best model, and it had a significant effect on the temporal change in abundance of this vole species. The GAMM smooth spline showed a non‐linear effect of years (edf = 3.157, *χ* ^2^ = 25.697, *p* < 0.001), reaching a significant positive peak in 2014, followed by a decreasing trend (Figure 10B). Conversely, the random effect for locations was not significant (edf = 0.001, *χ* ^2^ = 0.001, *p* = 0.642). The marginal effect analysis showed a significant positive slope in the increasing trend of the function between 2005 and 2012 (slope: 0.10–0.16, *z* = 2.22–2.85, *p* < 0.05) and a significant negative slope in the decreasing trend between 2016 and 2023 (slope: (−0.36)–(−0.11), *z* = 2.02–3.78, *p* < 0.05) (Figure 10C). The statistically significant slope intervals where water vole abundance showed significant change were marked on the partial conditional plot with the response scale (Figure 10D). The marginal effect contrast analysis of the nominal variable demonstrated a significant difference (GYB vs. DFP: difference = −1.37 ± 0.38 SE, *p* < 0.001).

**FIGURE 11:**
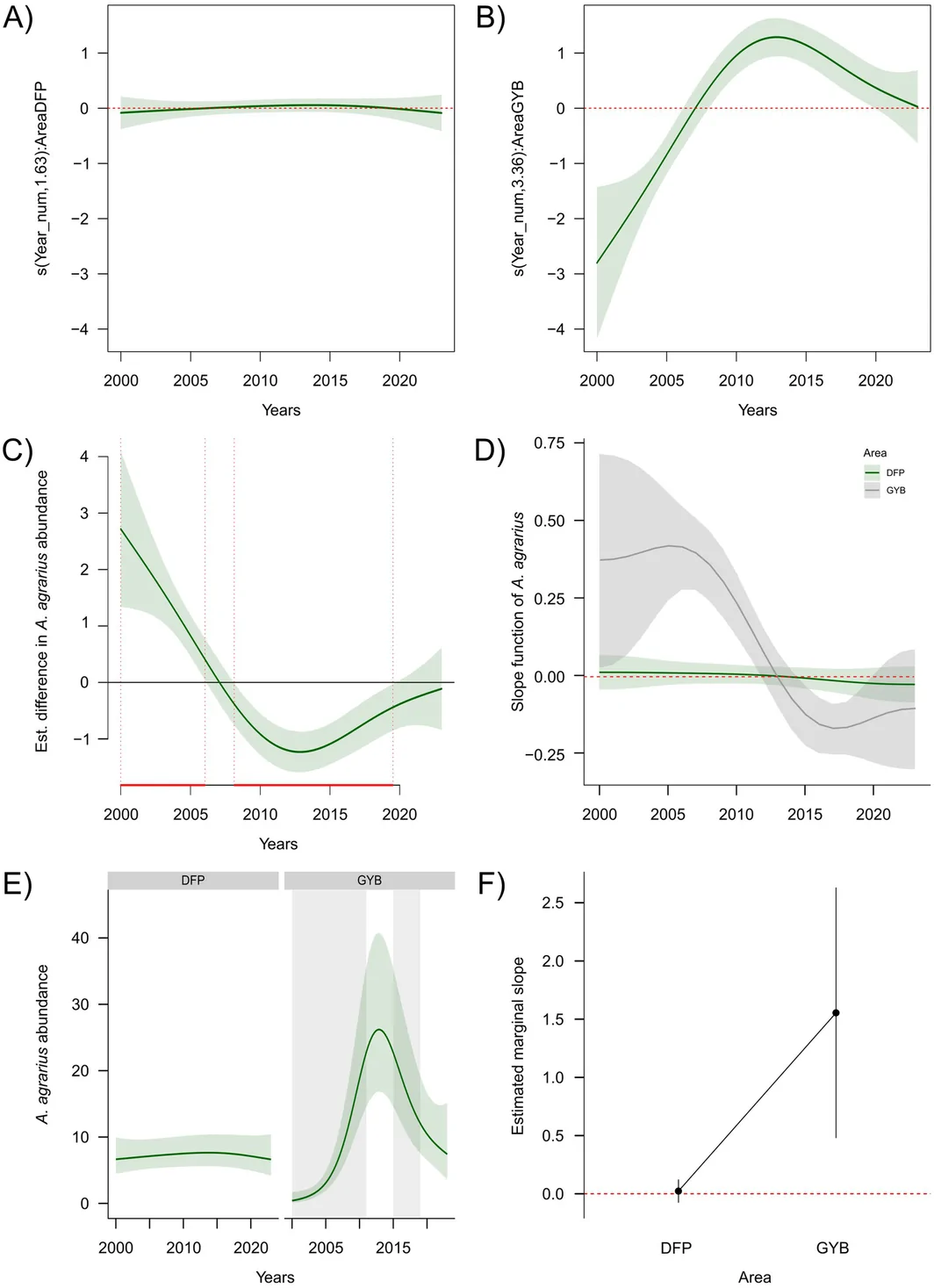
GAMM plots showing the effect of the interaction of years and the two investigated areas on the abundance of the striped field mouse ( *A. agrarius* ). (A) Smooth spline of years as a continuous explanatory variable in the case of the DFP. (B) Smooth spline of years as a continuous explanatory variable in the case of the GYB. (C) The smooth difference plot of the spline functions between two areas, the red lines indicate the periods when the abundance significantly differed between the two investigated areas. (D) Estimated slopes, where the red dashed line denotes a slope of zero (no change). (E) Nonlinear summed effect of years, where the vertical gray bars mark significant marginal effect (slope), showing the intervals in which the rate of change in the response variable was statistically confirmed. (F) Comparison of the slopes between the DFP and the GYB, where the red dashed line denotes a slope of zero.

The best candidate model for the striped field mouse (Aag ~ s(Year, by = Area) + s(Loc, bs = “re”) + offset(log_total); deviance explained 54.9%; *R* ^2^_adj_ = 0.670, REML = 591.010) demonstrated that the interaction of years and area significantly influenced the abundance change of this species. The smooth spline of the response variable showed a non‐linear effect in both areas, but the shapes of the function were different. Regarding the DFP, the smooth term was not significant, and the visualization of the smooth term for DFP confirmed the absence of a significant temporal trend (edf = 1.626, *χ* ^2^ = 0.767, *p* = 0.667) (Figure 11A). In contrast, the smooth term was significant in the case of the GYB (edf = 3.364, *χ* ^2^ = 57.746, *p* < 0.001). The smooth spline reached a significant positive peak in 2013, followed by a decreasing trend (Figure 11B). Based on the plot difference function, there were significant differences in the striped field mouse abundance between 2000–2006 and 2008–2020 (Figure 11C). The random effect for locations was significant (edf = 20.755, *χ* ^2^ = 148.403, *p* < 0.001).

The marginal effect analysis confirmed that the estimated slope was non‐significant in the entire sampling period in DFP (Figure 11D). In contrast, a significant positive slope in the increasing trend of the GYB partial GAMM function between 2000 and 2011 (slope: 0.15–0.42, *z* = 2.13–6.68, *p* < 0.05) and a significant negative slope in the decreasing trend between 2015 and 2019 (slope: (−0.17)–(−0.12), *z* = 2.54–4.08, *p* < 0.05) (Figure 11D). The statistically significant marginal effects were marked on the partial conditional plot in GYB (Figure 11E). In the case of the nominal variable (Area), the estimated marginal (average) slope of DFP was non‐significant positive (DFP: slope = 0.02, *z* = 0.45, *p* = 0.654), while that of the GYB was significant (GYB: slope = 1.55, *z* = 2.83, *p* = 0.005) (Figure 11F). The marginal effect contrast between the two areas was also significant for this species (GYB vs. DFP difference = 7.14 ± 1.8 SE, *z* = 3.98, *p* < 0.001).

**FIGURE 12:**
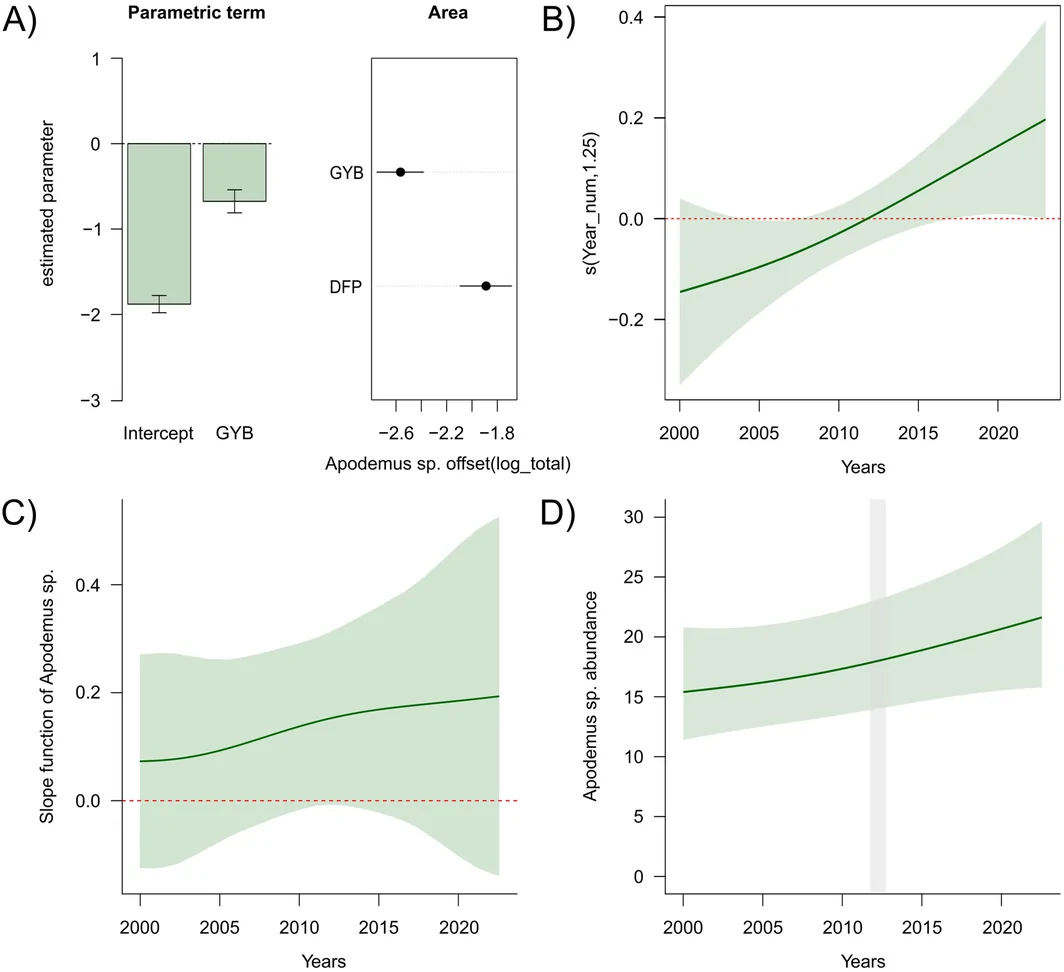
GAMM plots showing the effect of the two investigated areas and years on the abundance of the Sylvaemus group (*Apodemus* sp.). (A) Parametric term effect plots with the estimated coefficients of areas are shown on the left side, and their fitted effects are shown on the right side. (B) Smooth spline of years as a continuous explanatory variable. (C) Estimated slope, where the red dashed line denotes a slope of zero (no change). (D) Nonlinear summed effect of years, where the vertical gray bar marks significant marginal effect (slope), showing the intervals in which the rate of change in the response variable was statistically confirmed.

The best‐fitted candidate model for the Sylvaemus group ( *Apodemus flavicollis* , *A. sylvaticus* , *A. uralensis* ) included the main effect of the smooth term of year, the parametric effect of area and the random effect for breeding locations (Asp ~ s(Year) + Area + s(Loc, bs = “re”) + offset(log_total)). This model explained 22.6% of the total deviance (*R* ^2^_adj_ = 0.641, REML = 824.230). The importance of the Area effect was significant in this model (Area: *χ* ^2^ = 25.070, *p* < 0.001), and this Apodemus taxon was more abundant in the DFP (estimated parameter = −0.675 ± 0.135 SE, *z* = 5.007, *p* < 0.001) (Figure 12A). The partial effect plot of years showed an increasing non‐linear smooth spline, but this effect was not significant (edf = 1.249, *χ* ^2^ = 5.075, *p* = 0.066) (Figure 12B). The random effect for locations was also significant (edf = 8.505, *χ* ^2^ = 14.166, *p* = 0.034). The marginal effect analysis showed a significant positive slope between 2012 and 2013 (slope: 0.02, *z* = 2.00, *p* < 0.05) (Figure 12C). The statistically significant marginal effects were marked on the partial conditional plot with the response scale (abundance) (Figure 12D). In the case of the parametric term, the marginal effect contrast between the two areas was significant (GYB vs. DFP difference = −8.57 ± 1.91 SE, *z* = −4.48, *p* < 0.001).

**FIGURE 13:**
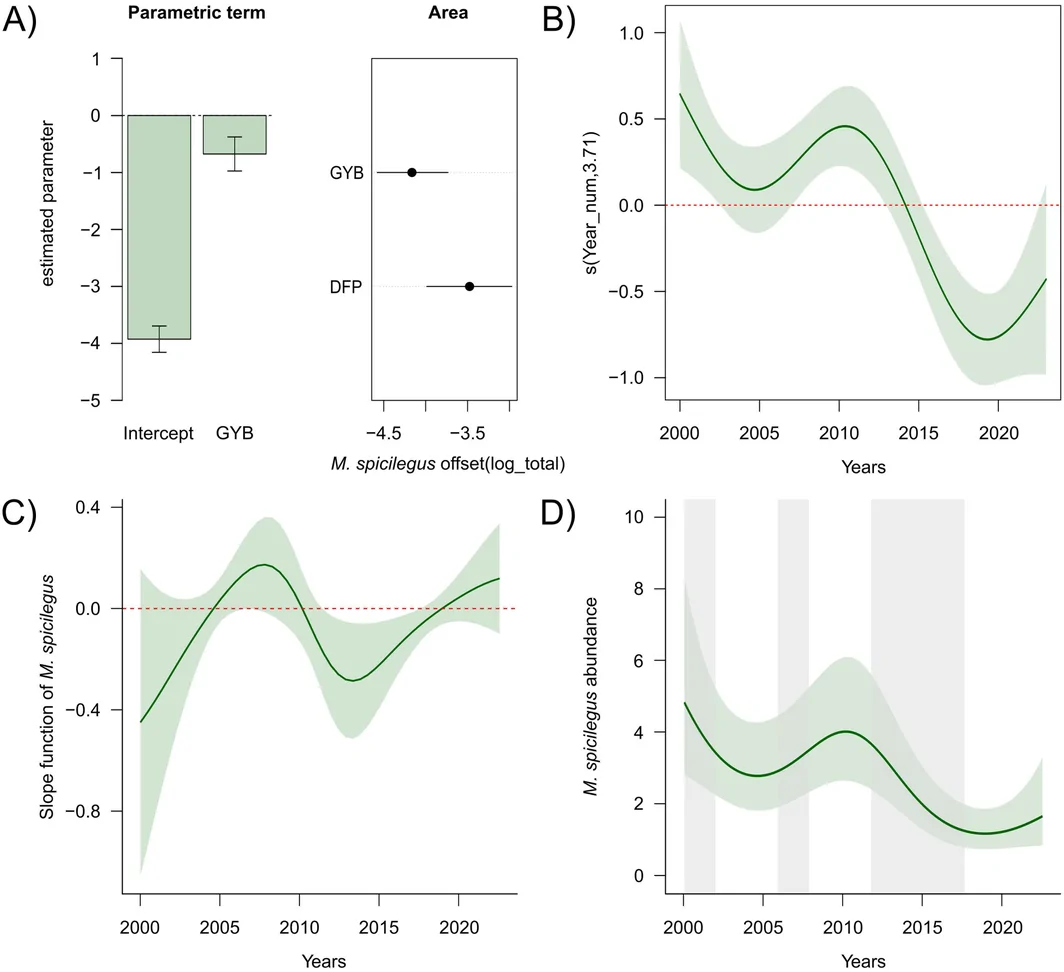
GAMM plots showing the effect of the two investigated areas and years on the abundance of the mound‐building mouse ( *M. spicilegus* ). (A) Parametric term effect plots with the estimated coefficients of areas are shown on the left side, and their fitted effects are shown on the right side. (B) Smooth spline of years as a continuous explanatory variable. (C) Estimated slope, where the red dashed line denotes a slope of zero (no change). (D) Nonlinear summed effect of years, where the vertical gray bars mark significant marginal effect (slope), showing the intervals in which the rate of change in the response variable was statistically confirmed.

Regarding the mound‐building mouse, the best model included the effects of the year and geographic area as well as the random effect for locations (Msp ~ s(Year) + Area + s(Loc, bs = “re”) + offset(log_total)). The model explained 34.5% of the deviance (*R* ^2^_adj_ = 0.576, REML = 450.140). The effect of Area as nominal predictor was significant in this candidate model (*χ* ^2^ = 5.112, *p* = 0.024). The parametric term showed significant results, according to which the abundance of the mound‐building mouse in the DFP was significantly higher than in the GYB (estimated parameter = −0.676 ± 0.299 SE, z = 2.261, *p* = 0.024) (Figure 13A). Based on the results of the smooth term, the years had a significant effect on the temporal change in abundance of this mouse species. The GAMM smooth spline showed a non‐linear fluctuating and decreasing effect of years (edf = 3.708, *χ* ^2^ = 34.980, *p* < 0.001) (Figure 13B). The random effect for locations was also significant (edf = 14.643, *χ* ^2^ = 44.050, *p* < 0.001). The marginal effect analysis showed a significant negative slope between 2000 and 2002 (slope: (−0.18)–(−0.15), *z* = 2.25–2.43, *p* < 0.05), a significant positive slope between 2006 and 2008 (slope: 0.07–0.10, *z* = 2.01–2.38, *p* < 0.05), and another significant negative slope between 2012 and 2018 (slope: (−0.22)–(−0.10), *z* = 2.57–4.94, *p* ≤ 0.01) (Figure 13C). The partial conditional plot with the response scale (abundance) shows the intervals in which marginal effects were statistically significant (Figure 13D). The marginal effect contrast value between the two areas was slightly above the significance threshold (GYB vs. DFP difference = −1.89 ± 0.98 SE, *z* = −1.94, *p* = 0.053).

In the next part of this scenario, the effect of the weather conditions was analyzed by GAMM models. For each prey taxon selected by the “mvabund” framework, two alternative weather models were fitted. The first model included the observed temperature (T) and rainfall (R) as linear predictors, whereas the second model included the corresponding deviation variables (dev(T) and dev(R)), representing departures from the long‐term average conditions. Model selection was based on the comparison of these alternative formulations, and the model including the observed weather variables (T and R) was retained because it provided a better fit for the selected taxa. The significant multivariate Wald test indicated that prey composition was influenced by weather parameters (T: *χ* ^2^ = 10.08, *p* < 0.001; R: *χ* ^2^ = 11.22, *p* < 0.001).

Taxon‐specific Wald tests showed significant effects for the following taxa: *Sorex araneus* (Sar; *χ* ^2^ = 6.590, *p* < 0.001), *Sorex minutus* (Smi; *χ* ^2^ = 5.329, *p* < 0.001), *Crocidura suaveolens* (Csu; *χ* ^2^ = 4.271, *p* = 0.011), and *Apodemus* spp. (Asp; *χ* ^2^ = 5.466, *p* < 0.001). No significant T + R effect was detected for the remaining taxa (*p* > 0.05).

**FIGURE 14:**
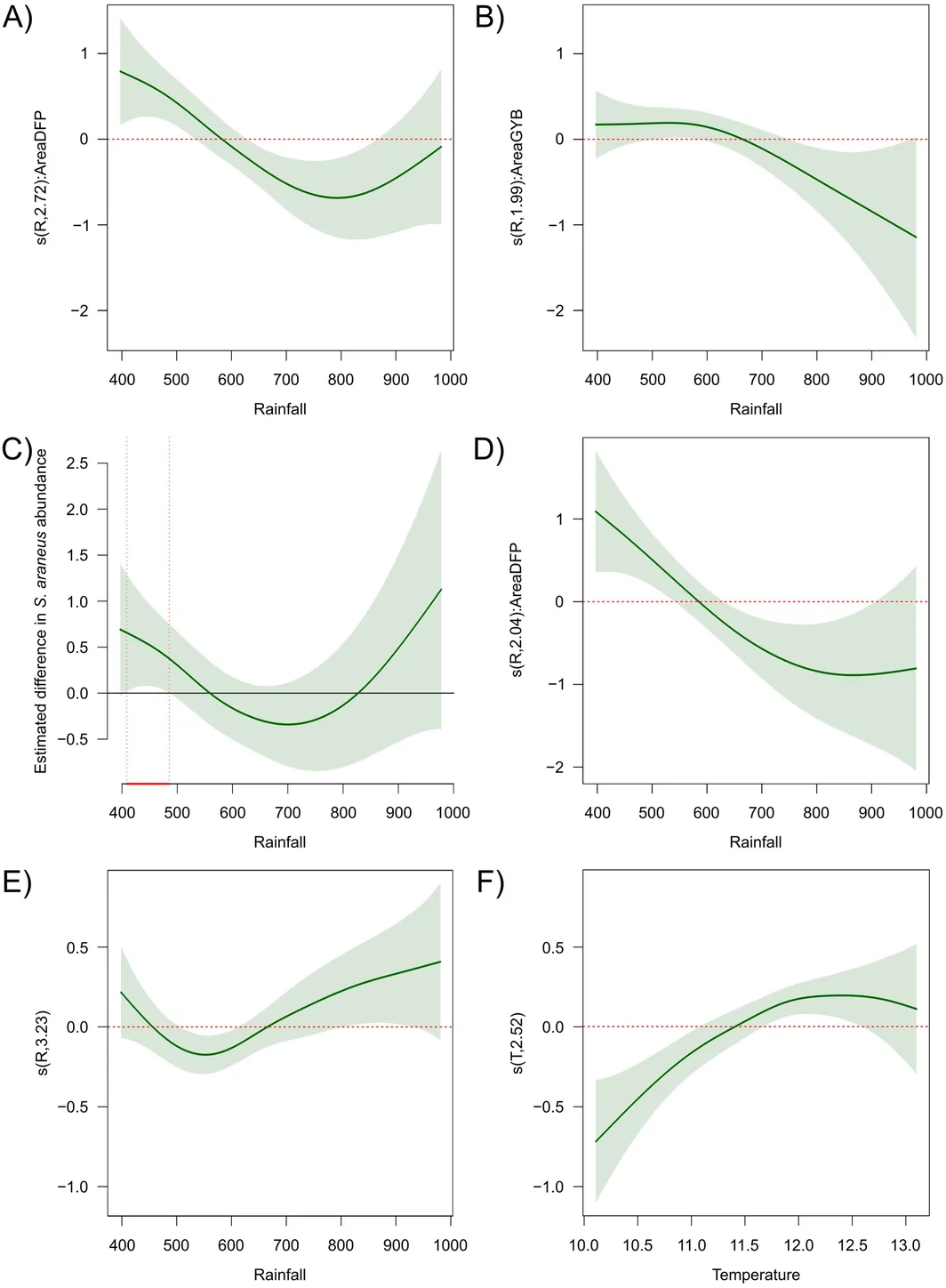
GAMM plots showing the effect of the weather conditions and the two investigated areas. (A) Effects of rainfall as a continuous explanatory variable on the abundance of the common shrew ( *S. araneus* ) in the DFP, fitted with a smooth spline. (B) Effects of rainfall as a continuous explanatory variable on the abundance of the common shrew ( *S. araneus* ) in the GYB, fitted with a smooth spline. (C) The smooth difference plot of the spline functions between two areas, the red lines indicate the amount of rainfall where the abundance of the common shrew significantly differed between the two investigated areas. (D) Effects of rainfall as an explanatory variable on the abundance of the pygmy shrew ( *S. minutus* ) in the DFP, fitted with a smooth spline. (E) Smooth spline of rainfall as a continuous explanatory variable affecting the Sylvaemus group (*Apodemus* sp.) abundance. (F) Smooth spline of temperature as a continuous explanatory variable affecting the Sylvaemus group (*Apodemus* sp.) abundance.

In the case of the common shrew, the better candidate GAMM model included the interaction of the two weather conditions and geographic area, and the localities as a random effect (Sar ~ s(R, by = Area) + s(T, by = Area) + s(Loc, bs = “re”) + offset(log_total)), which model explained 60.8% of the total deviance (*R* ^2^_adj_ = 0.853, REML = 661.490). The smooth spline of the response variable showed a significant non‐linear effect in both areas, but the shapes of the function were different. The visualization of the smooth term for DFP confirms a significant temporal trend; the spline reached a significant negative low point around 800 mm of rainfall, followed by an increasing trend (edf = 2.720, *χ* ^2^ = 15.274, *p* = 0.002) (Figure 14A). In the case of the GYB, we showed an opposite effect, it stagnated until about 580 mm, after which a decreasing trend was observed (edf = 1.995, *χ* ^2^ = 7.036, *p* = 0.047) (Figure 14B). Based on the plot difference function, there were significant differences in the common shrew abundance between 408.798–485.485 mm (Figure 14C). Temperature had no significant effect in any area (DFP: edf = 2.264, *χ* ^2^ = 1.775, *p* = 0.480; GYB: edf = 1.001, *χ* ^2^ = 0.118, *p* = 0.732). The random effect of locations as a smooth term was significant (edf = 22.568, *χ* ^2^ = 191.187, *p* < 0.001). Regarding the marginal effect analysis, the estimated slope of the partial GAMM function showed significant negative values in both areas within a single rainfall interval (DFP: 505.15–634.93 mm; GYB: 699.82–764.70 mm). The estimated marginal effect was significant in DFP (slope = −0.04 ± 0.02 SE, *z* = −2.00, *p* = 0.046) and not significant in GYB (slope = −0.02 ± 0.01 SE, *z* = −1.19, *p* = 0.233). The marginal effect contrast value between the two areas was slightly above the significance threshold (GYB vs. DFP difference = −0.0032 ± 0.0026 SE, *t* = −1.194, *p* = 0.234). The better candidate model for the pygmy shrew also included the interaction of the two weather conditions and geographic area, and the localities as a random effect (Smi ~ s(R, by = Area) + s(T, by = Area) + s(Loc, bs = “re”) + offset(log_total)), which model explained 46.7% of the total deviance (*R* ^2^_adj_ = 0.817, REML = 433.500). The smooth spline of the response variable showed a significant non‐linear effect only in the case of the DFP. Based on the visualization of the smooth term, the spline decreased to about 850 mm, after which a slight increase can be observed (edf = 2.043, *χ* ^2^ = 12.317, *p* = 0.004) (Figure 14D). In the case of the GYB, the smooth spline of the response variable was not significant (edf = 1.000, *χ* ^2^ = 0.661, *p* = 0.416). Temperature did not affect the abundance of this shrew species in any of the investigated areas (DFP: edf = 1.160, *χ* ^2^ = 1.281, *p* = 0.279; GYB: edf = 1.000, *χ* ^2^ = 2.398, *p* = 0.122). The random effect of locations as a smooth term was significant (edf = 19.064, *χ* ^2^ = 83.045, *p* < 0.001). The marginal effect analysis demonstrated that the estimated slope of the partial GAMM function had significant negative values in DFP in one rainfall interval (DFP: 483.52–656.56 mm), while no significant slope values were estimated in GYB. The marginal effect was significant in DFP (slope = −0.01 ± 5.47e‐3 SE, *z* = −2.26, *p* = 0.024) and not significant in GYB (slope = −2.06e‐3 ± 2.48e‐3 SE, *z* = −0.83, *p* = 0.405). The marginal effect contrast value between the two areas was slightly above the significance threshold (GYB vs. DFP difference = −0.005 ± 0.003 SE, *t* = −1.930, *p* = 0.055). For the lesser white‐toothed shrew, the better candidate model included the two weather parameters as smooth terms, and the localities as a random effect (Csu ~ s(R) + s(T) + s(Loc, bs = “re”) + offset(log_total)). This model explained 44.5% of the total deviance (*R* ^2^_adj_ = 0.823, REML = 542.620). The amount of rainfall had no effect on the abundance change of this shrew species (edf = 1.000, *χ* ^2^ = 2.351, *p* = 0.125), and temperature did not significantly influence the abundance change (edf = 1.000, *χ* ^2^ = 1.518, *p* = 0.218). However, the random effect of locations as a smooth term was significant (edf = 19.750, *χ* ^2^ = 128.044, *p* < 0.001). In the case of the Sylvaemus group ( *Apodemus flavicollis* , *A. sylvaticus* , *A. uralensis* ), the better candidate model included the two weather parameters as smooth terms, and the localities as a random effect (Asp ~ s(R) + s(T) + s(Loc, bs = “re”) + offset(log_total)), which explained 34.4% of the total deviance (*R* ^2^_adj_ = 0.741, REML = 825.960). The amount of rainfall had a significant effect on the abundance change of this Apodemus taxon (edf = 3.229, *χ* ^2^ = 10.860, *p* = 0.025) (Figure 14E), and temperature also significantly influenced the abundance change (edf = 2.515, *χ* ^2^ = 20.29, *p* < 0.001) (Figure 14F). The random effect of locations as a smooth term was significant (edf = 17.680, *χ* ^2^ = 59.050, *p* < 0.001). According to the marginal effect analysis of rainfall, the estimated slope of the partial GAMM function showed significant negative values within a single rainfall interval (440.26–461.89 mm). The estimated marginal effect was not significant (slope = 0.01 ± 0.01 SE, *z* = 0.95, *p* = 0.341). The estimated slope of the temperature effect showed significant positive estimated values in one temperature interval (10.11°C–11.66°C). The estimated marginal effect of temperature was significant (slope = 5.20 ± 1.72 SE, *z* = 3.03, *p* = 0.002).

## 4 Discussion

Small mammals (rodents and shrews) play a key role in several ecosystems all over the world (Feldhamer 2015), for example, they are an important part of the food chain for many predators (e.g., Askew et al. 2007; Cully Jr. et al. 2010; Korpimäki and Norrdahl 1991; Matos et al. 2015; Paspali et al. 2013; Spencer et al. 2014; Virgós et al. 1999). In this paper, small mammal assemblages were evaluated based on pellet analysis in two different lowland regions of Hungary. We investigated the abundance of the main and alternative prey species, as well as the change of diversity metrics and community dynamic characteristics over time. In general, pellets can be collected in large numbers (Taylor 2004), and it is a highly efficient method of monitoring small mammal distribution, population dynamics, and community composition (Heisler et al. 2016; Stefke and Landler 2020; Torre et al. 2015). Western Barn Owls consume many potential alternative prey according to their availability (Berg and Ille 2002; Janžekovič and Klenovšek 2020; Moysi et al. 2018; Romano et al. 2020; Tores et al. 2005), but they feed primarily on nocturnal, terrestrial small mammal species (e.g., Horváth et al. 2018, 2020; Milana et al. 2016; Milchev 2015; Szép et al. 2021; Taylor 2004; Torre et al. 2015). Our results are in accordance with this finding because more than 99% of the food composition was small mammals.

Western Barn Owls preyed on the common vole in the highest proportion in both areas; however, in the DFP, this prey species made up a significantly larger part of the diet composition. The importance of common voles in the diet of owls has been demonstrated in numerous studies (e.g., Bernard et al. 2010; Frey et al. 2011; Horváth et al. 2018, 2020, 2022; Horváth, Maurer, and Horváth 2023; Kitowski 2013; Petrovici et al. 2013; Purger 2014; Veselovský et al. 2017). Prey selection is influenced by habitat characteristics, weather conditions, and prey availability (Granjon and Traoré 2007; McDowell and Medlin 2009; Milchev 2015); thus, the diet composition of owls varies between different regions (e.g., González‐Fischer et al. 2011; Moysi et al. 2018; Obuch and Khaleghizadeh 2011; Riegert et al. 2021; Romano et al. 2020) and changes over time (e.g., Horváth et al. 2020; Love et al. 2000; Meek et al. 2012; Milana et al. 2018; Szép et al. 2021). Earlier studies demonstrated that along the Drava River, especially in the DFP, wood mice (*Apodemus* species) are important alternative prey (Horváth et al. 2005, 2020, 2024; Purger 2014; Szép et al. 2017; Szűcs et al. 2014), and the Crocidura shrews also occurred with high abundance in the food composition of the Western Barn Owls (Szűcs et al. 2014). In contrast, the common shrew was identified as the main alternative prey in the GYB (Szűcs et al. 2014). Our results supported the alternative prey selection of Western Barn Owls because the proportion of the *Apodemus* and *Crocidura* species was significantly higher in the DFP, and the common shrew occurred at a higher frequency in the GYB. The importance of the wood mice as alternative prey has been described in several studies (Bontzorlos et al. 2005; Pezzo and Morimando 1995; Rodríguez and Peris 2007). Crocidura species occur in dry, open, semi‐natural habitats and cultivated landscapes (Anděra and Horáček 2005; Heroldová et al. 2007; Nowak 1999; Poláčiková 2010; Szép et al. 2018; Varuzza et al. 2001) and also indicate drier weather periods (Herczeg and Horvath 2015). Thus, the higher occurrence of the bicolored shrew ( *C. leucodon* ) and lesser white‐toothed shrew ( *C. suaveolens* ) may reflect that these dry habitats are typical of the owls' hunting areas (Bosé and Guidali 2001; Obuch and Khaleghizadeh 2011; Paspali et al. 2013). Some earlier studies showed that the abundnace of the Crocidura genus was higher in the drier lowland regions of Hungary (Schmidt 1969; Szűcs et al. 2014), which is supported by our results. In contrast, the percentage distribution of the common shrew ( *Sorex araneus* ) abundance was significantly higher in the GYB, and it was the second most preyed species, which reflected that the common shrew is a typical alternative prey for the Western Barn Owls in this region. Unlike in the DFP, there are many marshlands in the GYB, and the common shrew is associated with a wide variety of habitat types (Krištofík 2012; Schlinkert et al. 2016; Sundell et al. 2012; Tattersall et al. 2002; Wang and Grimm 2007), including these moist habitats (Ambros et al. 1999; Dudich et al. 1985; Dudich and Štollmann 1988; Krištofík 2001, 2012). Our results suggest that the common shrew represents an important component of the prey assemblage associated with marshland habitats in the owls' hunting areas.

Several studies estimated the species richness and the diversity of small mammal assemblages based on owl pellet analysis (e.g., Balestrieri et al. 2019; González‐Fischer et al. 2012; Horváth et al. 2022; Massa et al. 2014; Milana et al. 2019; Millán de la Peña et al. 2003; Stefke and Landler 2020; Torre et al. 2015; Vigués et al. 2018) since the diet of Western Barn Owls, as opportunistic predators, provides a reliable proxy for the composition of accessible small mammal assemblages within their hunting areas (Ferri et al. 2021; Horváth et al. 2022; Onduso et al. 2025; Taylor 2004; Tores et al. 2005). The rarefaction analysis showed a significant difference in species richness, Shannon diversity and Simpson dominance between the two lowland areas. The diversity indices of the Western Barn Owl's diet were significantly higher in the GYB. This finding may be caused by the different weather conditions or landscape structure of the two lowland areas. In Italy, the spatial change of the small mammal assemblages was investigated based on owl pellet analysis, and the paper pointed out that the diversity was different in the comparison of the study sites, which was caused by the intensification of agriculture in certain regions (Milana et al. 2016). Based on this indirect method, it was also examined in Spain how human‐induced landscape changes affected the small mammal community (Vigués et al. 2018). Meteorological variables may affect landscape structure and productivity, thereby modifying food availability and refuge for small mammals; thus, the abundance of these species and the composition of the communities in a given area are also influenced by weather (Ernest et al. 2000; Milstead et al. 2007).

Based on the results of the PERMANOVA, the significant separation between the two areas highlights that the diet of Western Barn Owls is different across these landscapes. However, as indicated by the rank‐curve analysis, this separation mainly reflects differences in the relative abundances of shared prey species rather than species replacement between the two areas. The narrow clustering of the DFP samples in the PCoA scatter plot suggests that the diet is dominated by high‐density prey species, such as the common vole ( *Microtus arvalis* ). In contrast, the wider multivariate dispersion observed in the GYB reflects a higher inter‐nesting pair or inter‐sampling location dietary variation. These results supported our previous observation (Horváth, Maurer, and Horváth 2023), which shows that the niche breadth and individual‐level niche overlap of Western Barn Owls differ significantly between these two investigated areas. In this current study, the lower dietary diversity and narrow PCoA clustering of the DFP population indicate a lower niche breadth. In contrast, the higher small mammal prey diversity and wider multivariate dispersion in the GYB imply a higher niche breadth, where individual nesting pairs exploit different food resources.

Our above‐mentioned findings were also supported by the results of the rank‐curve analysis. The species difference value (Sp.D. = 0) indicated that no species substitutions occurred between the two assemblages. Therefore, the observed differences were primarily driven by changes in the relative abundances and ranking of shared prey taxa rather than by species turnover. Nevertheless, the low rank difference and the species difference values showed that the composition of the small mammal prey assemblages represented in the owl diet is broadly similar.

The landscape features and the weather conditions have an effect on small mammal communities and populations not only over a spatial scale but also over time. Nonetheless, besides these, many factors play a role in population dynamics; and the understanding of these mechanisms and how they affect each other (multiple‐factor hypothesis) is poorly understood (Andreassen et al. 2021; Krebs 2013). The GAMM results also supported the differences in species richness, Shannon diversity, and Simpson dominance between the two areas, as all three diversity indicators changed differently during the studied period.

The community dynamic characteristics (rank change, rank shift, and turnover) of small mammal assemblages were also examined in relation to the years and the two areas. Based on our GAM results, there were significant temporal and spatial changes in rank change, but not in rank shift and turnover, which means that the relative abundance of prey species represented in the owl diet changes in the spatiotemporal scale. Small mammal community changes are already known phenomena driven by several biotic and abiotic factors. One of the most researched of these is climate change, which affects communities not only directly but also indirectly. Meserve et al. (2011) described that increased rainfall due to El Niño Southern Oscillations (ENSOs) changes small mammal assemblage structure in north‐central Chile. Global warming can also indirectly affect animal populations through habitat changes, and the varying land‐use practices, as well as other anthropogenic activities, also contribute to structural changes in communities (Moritz et al. 2008; Parsons et al. 2013; Presley et al. 2019; Zárybnická et al. 2017). Jones et al. (2017) also studied small mammal communities, among other animal taxa, and they explored that species abundances and community composition might be a better indicator of the community response to environmental change. Thus, the investigation of community dynamic characteristics is an important subject to understand the community‐level response of small mammals to a changing abiotic factor and how it influences biotic factors.

The GAMM results supported the above‐mentioned findings that the abundance of the prey taxa differed in the two lowland regions. Among the shrew species, the lesser white‐toothed shrew occurred with higher frequency in the DFP; in contrast, the Sorex species are more abundant in the GYB. There were also differences in abundance among rodents. The populations of European water vole declined across Europe over the past half century due to the habitat loss and fragmentation (Lawton and Woodroffe 1991; Rushton et al. 2000; Strachan 2004). With the disappearance and transformation of wetlands, water voles have appeared in grasslands and farmlands (Delattre et al. 2006; Halliez et al. 2015; Wijas et al. 2023). Besides these, the appearance and expansion of the American mink (*Neogale vison*) as a potential predator in Europe also contributed to the decline in vole populations (Barreto and Macdonald 2000; Halliwell and Macdonald 1996; Rushton et al. 2000; Woodroffe et al. 1990). The species has only appeared in Hungary in recent years, mainly in the Szigetköz (Kanizsay et al. 2025), which is part of the GYB included in our study. Based on our results, this population decline can also be observed in Hungary. We also supported Szűcs et al.'s (2014) result, according to which water voles occur more frequently in the DFP. The reason for the difference is that although both regions are lowland and have a significant surface water network, the land use and soil conditions are radically different. In the GYB, the agricultural landscape is characterized by extreme homogeneity and patches of inland water and bound soil remaining after floods, which are not conducive to water voles; thus, this species only occurs in populations restricted to narrow canal banks. In contrast, small‐scale agriculture is more widespread in the DFP, where deep plowing is less common. And the extensive backwater system of this area provides favorable conditions, as well as the clay and sandy soils found in the area make it easy to create a burrow system. The GAMM results support the fact, the *Apodemus* species (striped field mouse and Sylvaemus group) were a significant alternative prey in the DFP. Regarding the striped field mouse, the abundance change during the investigated period confirmed that this species is a constant but nondominant prey in the DFP, which is consistent with findings from other publications (Horváth et al. 2005; Purger 2014; Szép et al. 2017). This species is not only a significant alternative prey in the southern Transdanubia, but also an important element of the diet in the northwestern region of the country. In the mid‐1900s, it only occurred in areas south of Lake Balaton (Schmidt 1976), but in recent decades its northward spread has been observed (Bihari 2007), as a result of which it has also appeared in areas north and northwest of Hungary, such as Austria and Slovakia (Herzig‐Straschil et al. 2003; Obuch et al. 2016; Tulis et al. 2016). Temporal change in the species in the GYB based on GAMM supports the northwestern expansion of this species.

In the case of the mound‐building mouse, it occurs in a higher amount in the diet of owls in the DFP based on the GAMM results. This species is distributed from Russia (Caucasus) to the Carpathian Basin, and it has isolated populations on the Balkan Peninsula (Bonhomme and Guénet 1996; Boursot et al. 1993; Marshall and Sage 1981; Mitchell‐Jones et al. 1999; Sage et al. 1993). The mound‐building mouse is distributed in grassland, steppe, and cultivated land (Coroiu et al. 2016). In Hungary, it is highly abundant in the lowland regions, particularly across the Great Hungarian Plain and the Tiszántúl region, as well as in Somogy County; and it inhabits open, steppe‐like habitats, such as agricultural edges and fallow lands (Bihari 2007). Based on our results, this species occurs in higher abundance in the DFP, which is due to the fact that, on the one hand, the marginal zone of the species' distribution is the northwestern part of the Carpathian Basin (such as the GYB), where the populations are much rarer; and on the other hand, the climate and the soil characteristics necessary for mound‐building are more favorable. Our results also highlighted that the species' population change is characterized by a decreasing trend, which supported the fact that the loss of steppe grassland and the change in agricultural cultivation lead to population decline (Coroiu et al. 2016).

The results of the generalized additive mixed models (GAMMs) testing the effect of weather parameters showed that annual rainfall is an important factor driving the population of both common and pygmy shrews, while temperature is irrelevant. The impact of various weather factors on Sorex species abundance has been described in several studies (Churchfield et al. 1995; Dokulilová et al. 2023; Hlôška et al. 2016; Ivanter 2020; Stefke and Landler 2020; Zárybnická et al. 2017; Zub et al. 2012). Because of their fast metabolism (Churchfield 1990; Taylor 1998, 2023), these small mammals are incredibly sensitive to moisture levels, which directly affects the amount of food available to them (Churchfield 1982; Ivanter 2020). However, how their reaction to rainfall depends completely on the local landscape, leading to differences between the two investigated areas. In the DFP, both species showed a significant, non‐linear relationship with rainfall. Their number decreased with increasing precipitation, but above 900 mm of rain their number increased again, probably because local flooding forced them into the remaining dry spots, or it may also partially represent a statistical edge effect of smooth splines. On the other hand, in the heavily modified (river regulation and flood protection, agricultural development) GYB, shrew numbers decreased dramatically once rainfall went over 580 mm. Heavy rain in the GYB causes serious waterlogging, which floods the underground tunnels that common shrews use for nesting and hunting.

Our results indicate that the abundance dynamics of the lesser white‐toothed shrew ( *Crocidura suaveolens* ) did not depend on weather conditions. Although this species prefers a dry, warmer climate (Nowak 1999) and indicates drier weather periods (Herczeg and Horvath 2015), neither rainfall nor temperature had a significant influence on the abundance. Similar results were obtained by Torre et al. (2020) for the greater white‐toothed shrew ( *C. russula* ) in Spain, where increasing temperatures and decreasing precipitation did not explain the change in shrew numbers.

Our result demonstrates that the abundance of the Sylvaemus group ( *Apodemus flavicollis* , *A. sylvaticus* , *A. uralensis* ) is influenced by the weather conditions, both rainfall and temperature exerted a significant, non‐linear effect. These climatic drivers affect the abundance of *Apodemus* species through both primary productivity and various physiological processes. Regarding productivity, moderate rainfall acts as a bottom‐up driver by enhancing Quercus and Fagus growth (Čepelka et al. 2020; Díaz et al. 2010; Marini et al. 2023; Šipoš et al. 2017), while extreme heat destroys vegetation needed for shelter (Santoro et al. 2017). Physiologically, moderate rain extends breeding and increases reproductive success (Andreassen et al. 2021; Díaz et al. 2010), but extreme rain floods burrows, causing mortality and reduces feeding efficiency (Andreassen et al. 2021).

Our GAMM results confirm that the abundance of small mammal taxa represented in the owl diet may differ in different geographical areas, depending on their characteristics. Stefke and Landler (2020) analyzed rodent and shrew community composition and abundance at Lake Fertő in Eastern Austria. The research showed that there was a significant change in rodent and shrew abundance, caused by abiotic factors such as temperature and precipitation. In addition, the research raises the possibility that the drainage efforts may also contribute to this change. Other land use modifications can also cause changes in population dynamics, such as the intensification of agriculture or the use of chemicals, which were also described across Europe (Battisti et al. 2020; Love et al. 2000; Milana et al. 2018).

Although Western Barn Owl pellets provide an efficient and widely accepted proxy for assessing small mammal assemblages, the observed prey composition reflects the accessible prey community filtered by owl hunting behavior, prey detectability, habitat use, and prey‐specific vulnerability. Therefore, our interpretations refer to the prey assemblages represented in the owl diet rather than to an unbiased census of the entire small mammal community.

## 5 Conclusion

Our results demonstrate that Western Barn Owl pellet analysis provides a valuable long‐term proxy for assessing spatial and temporal variation in accessible small mammal assemblages. Prey composition, diversity, and community dynamics differed markedly between the two lowland regions, indicating that local landscape characteristics and weather conditions jointly shape the prey assemblages available to hunting owls. Moreover, the effects of weather variables were taxon‐specific and differed between regions, highlighting that the responses of small mammals to climatic conditions depend on local environmental context. These findings emphasize the usefulness of long‐term pellet monitoring for detecting community‐level changes in small mammal assemblages under ongoing environmental and climate change, while acknowledging that the inferred patterns reflect the prey community represented in the diet of Western Barn Owls rather than the entire small mammal community.

## Author Contributions

**Győző F. Horváth:** conceptualization (lead), formal analysis (equal), methodology (lead), supervision (lead), writing – original draft (equal), writing – review and editing (equal). **Gábor Takács:** investigation (equal), project administration (equal), writing – review and editing (equal). **Dániel Molnár:** investigation (equal), project administration (equal), writing – review and editing (equal). **Adrienn Horváth:** data curation (lead), formal analysis (equal), methodology (supporting), visualization (lead), writing – original draft (equal), writing – review and editing (equal).

## Funding

The authors have nothing to report.

## Consent

All authors have given consent to publish.

## Conflicts of Interest

The authors declare no conflicts of interest.

## Supporting information


**Appendix S1:** Sampling data and total numbers of small mammal prey items in the two investigated areas (MNI: minimum number of individuals) (Author's Work).

**Appendix S2:** The minimum number of individuals (MNI) of small mammal taxa selected with the manyglm() function in R (in brackets the percentage frequency of occurrence, %MNI) (Author's Work).

## Data Availability

The authors declare that the data supporting the findings of this study are available within the manuscript (Table 1, Appendices S1 and S2), and further inquiries can be directed to the corresponding author.
