# Supplementary material for: Long‐Term Temporal Change of Small Mammal Composition and Abundance in the Food Intake of the Western Barn Owl ( Tyto alba ), Comparing Two Different Lowland Mesoregions
Source: Ecol Evol. 2026 Sep 11;16(9):e74309. doi: 10.1002/ece3.74309 (PMC13569878; doi:10.1002/ece3.74309)
Supplement: Supplementary file 1 [file ECE3-16-e74309-s001.docx]

**Appendix S2** The minimum number of individuals (MNI) of small mammal taxa selected with the manyglm() function in R (in brackets the percentage frequency of occurrence, %MNI) (Author's Work)

| Area | DFP | | | | | | |  | GYB | | | | | | |
| --- | --- | --- | --- | --- | --- | --- | --- | --- | --- | --- | --- | --- | --- | --- | --- |
| Year | Sar | Smi | Csu | Aam | Aag | Asp | Msp |  | Sar | Smi | Csu | Aam | Aag | Asp | Msp |
| 2000 | 42 (5,41%) | 14 (1,80%) | 21 (2,70%) | 7 (0,90%) | 89 (11,45%) | 177 (22,78%) | 41 (5,28%) |  | 60 (10,64%) | 10 (1,77%) | 1 (0,18%) | 0 (0,00%) | 0 (0,00%) | 74 (13,12%) | 32 (5,67%) |
| 2001 | 98 (4,89%) | 51 (2,54%) | 130 (6,48%) | 8 (0,40%) | 163 (8,13%) | 230 (11,47%) | 69 (3,44%) |  | 41 (22,40%) | 23 (12,57%) | 3 (1,64%) | 0 (0,00%) | 0 (0,00%) | 17 (9,29%) | 5 (2,73%) |
| 2002 | 15 (1,17%) | 6 (0,47%) | 43 (3,35%) | 12 (0,94%) | 45 (3,51%) | 165 (12,87%) | 29 (2,26%) |  | 13 (5,12%) | 4 (1,57%) | 20 (7,87%) | 0 (0,00%) | 0 (0,00%) | 15 (5,91%) | 0 (0,00%) |
| 2003 | 40 (8,77%) | 7 (1,54%) | 38 (8,33%) | 2 (0,44%) | 7 (1,54%) | 36 (7,89%) | 13 (2,85%) |  | 14 (5,15%) | 3 (1,10%) | 2 (0,74%) | 1 (0,37%) | 0 (0,00%) | 13 (4,78%) | 1 (0,37%) |
| 2004 | 5 (0,62%) | 1 (0,12%) | 30 (3,70%) | 2 (0,25%) | 60 (7,4%) | 180 (22,19%) | 30 (3,7%) |  | 23 (13,77%) | 20 (11,98%) | 5 (2,99%) | 0 (0,00%) | 1 (0,60%) | 4 (2,40%) | 1 (0,60%) |
| 2005 | 7 (0,77%) | 3 (0,33%) | 40 (4,42%) | 2 (0,22%) | 75 (8,3%) | 83 (9,18%) | 28 (3,1%) |  | 44 (8,41%) | 18 (3,44%) | 8 (1,53%) | 1 (0,19%) | 2 (0,38%) | 25 (4,78%) | 4 (0,76%) |
| 2006 | 9 (0,82%) | 3 (0,27%) | 64 (5,84%) | 9 (0,82%) | 124 (11,32%) | 102 (9,32%) | 16 (1,46%) |  | 159 (15,65%) | 52 (5,12%) | 46 (4,53%) | 1 (0,10%) | 29 (2,85%) | 90 (8,86%) | 13 (1,28%) |
| 2007 | 39 (4,52%) | 12 (1,39%) | 50 (5,80%) | 14 (1,62%) | 63 (7,31%) | 105 (12,18%) | 37 (4,29%) |  | 75 (15,99%) | 12 (2,56%) | 10 (2,13%) | 0 (0,00%) | 0 (0,00%) | 75 (15,99%) | 17 (3,62%) |
| 2008 | 12 (1,56%) | 4 (0,52%) | 26 (3,37%) | 18 (2,33%) | 38 (4,93%) | 157 (20,36%) | 17 (2,2%) |  | 29 (11,89%) | 8 (3,28%) | 4 (1,64%) | 2 (0,82%) | 2 (0,82%) | 24 (9,84%) | 2 (0,82%) |
| 2009 | 37 (3,56%) | 11 (1,06%) | 35 (3,37%) | 10 (0,96%) | 57 (5,49%) | 243 (23,41%) | 37 (3,56%) |  | 666 (44,67%) | 224 (15,02%) | 8 (0,54%) | 3 (0,20%) | 38 (2,55%) | 75 (5,03%) | 13 (0,87%) |
| 2010 | 15 (2,20%) | 4 (0,59%) | 40 (5,86%) | 10 (1,46%) | 65 (9,52%) | 123 (18,01%) | 45 (6,59%) |  | 272 (47,22%) | 80 (13,89%) | 1 (0,17%) | 0 (0,00%) | 26 (4,51%) | 24 (4,17%) | 3 (0,52%) |
| 2011 | 50 (3,97%) | 31 (2,46%) | 72 (5,72%) | 19 (1,51%) | 115 (9,14%) | 215 (17,09%) | 38 (3,02%) |  | 103 (17,17%) | 14 (2,33%) | 2 (0,33%) | 10 (1,67%) | 51 (8,50%) | 46 (7,67%) | 20 (3,33%) |
| 2012 | 27 (2,75%) | 7 (0,71%) | 32 (3,26%) | 8 (0,81%) | 101 (10,29%) | 119 (12,12%) | 11 (1,12%) |  | 153 (14,8%) | 50 (4,84%) | 3 (0,29%) | 1 (0,10%) | 55 (5,32%) | 93 (8,99%) | 28 (2,71%) |
| 2013 | 4 (1,10%) | 1 (0,28%) | 54 (14,92%) | 2 (0,55%) | 19 (5,25%) | 51 (14,09%) | 3 (0,83%) |  | 104 (29,89%) | 8 (2,30%) | 2 (0,57%) | 19 (5,46%) | 7 (2,01%) | 33 (9,48%) | 11 (3,16%) |
| 2014 | 4 (0,75%) | 1 (0,19%) | 60 (11,28%) | 2 (0,38%) | 23 (4,32%) | 160 (30,08%) | 7 (1,32%) |  | 0 (0,00%) | 0 (0,00%) | 0 (0,00%) | 0 (0,00%) | 0 (0,00%) | 0 (0,00%) | 0 (0,00%) |
| 2015 | 26 (1,98%) | 6 (0,46%) | 173 (13,15%) | 11 (0,84%) | 132 (10,03%) | 165 (12,54%) | 11 (0,84%) |  | 96 (13,69%) | 31 (4,42%) | 24 (3,42%) | 3 (0,43%) | 37 (5,28%) | 65 (9,27%) | 7 (1,00%) |
| 2016 | 8 (0,83%) | 2 (0,21%) | 46 (4,78%) | 20 (2,08%) | 71 (7,37%) | 288 (29,91%) | 11 (1,14%) |  | 27 (47,37%) | 18 (31,58%) | 3 (5,26%) | 0 (0,00%) | 3 (5,26%) | 0 (0,00%) | 0 (0,00%) |
| 2017 | 7 (0,59%) | 4 (0,34%) | 23 (1,94%) | 30 (2,53%) | 107 (9,03%) | 293 (24,73%) | 19 (1,6%) |  | 1202 (22,26%) | 392 (7,26%) | 211 (3,91%) | 14 (0,26%) | 159 (2,94%) | 409 (7,57%) | 36 (0,67%) |
| 2018 | 10 (1,18%) | 1 (0,12%) | 32 (3,77%) | 15 (1,77%) | 97 (11,44%) | 196 (23,11%) | 14 (1,65%) |  | 70 (13,38%) | 4 (0,76%) | 16 (3,06%) | 5 (0,96%) | 19 (3,63%) | 68 (13,00%) | 5 (0,96%) |
| 2019 | 7 (1,34%) | 2 (0,38%) | 11 (2,10%) | 10 (1,91%) | 44 (8,4%) | 114 (21,76%) | 15 (2,86%) |  | 255 (42,86%) | 51 (8,57%) | 5 (0,84%) | 4 (0,67%) | 10 (1,68%) | 38 (6,39%) | 2 (0,34%) |
| 2020 | 1 (0,71%) | 0 (0,00%) | 2 (1,43%) | 0 (0,00%) | 12 (8,57%) | 19 (13,57%) | 1 (0,71%) |  | 222 (35,07%) | 47 (7,42%) | 12 (1,90%) | 1 (0,16%) | 26 (4,11%) | 45 (7,11%) | 1 (0,16%) |
| 2021 | 8 (1,18%) | 8 (1,18%) | 45 (6,66%) | 7 (1,04%) | 19 (2,81%) | 100 (14,79%) | 7 (1,04%) |  | 287 (44,77%) | 93 (14,51%) | 18 (2,81%) | 0 (0,00%) | 6 (0,94%) | 58 (9,05%) | 3 (0,47%) |
| 2022 | 0 (0,00%) | 0 (0,00%) | 50 (13,48%) | 4 (1,08%) | 48 (12,94%) | 90 (24,26%) | 8 (2,16%) |  | 72 (13,69%) | 27 (5,13%) | 16 (3,04%) | 0 (0,00%) | 8 (1,52%) | 156 (29,66%) | 9 (1,71%) |
| 2023 | 0 (0,00%) | 0 (0,00%) | 51 (12,62%) | 0 (0,00%) | 14 (3,47%) | 42 (10,4%) | 2 (0,5%) |  | 53 (6,02%) | 39 (4,43%) | 56 (6,36%) | 0 (0,00%) | 16 (1,82%) | 66 (7,50%) | 4 (0,45%) |
