# Supplementary material for: Long‐Term Temporal Change of Small Mammal Composition and Abundance in the Food Intake of the Western Barn Owl ( Tyto alba ), Comparing Two Different Lowland Mesoregions
Source: Ecol Evol. 2026 Sep 11;16(9):e74309. doi: 10.1002/ece3.74309 (PMC13569878; doi:10.1002/ece3.74309)
Supplement: Supplementary file 2 [file ECE3-16-e74309-s002.docx]

**Appendix S1** Sampling data and total numbers of small mammal prey items in the two investigated areas (MNI: minimum number of individuals) (Author's Work)

| Area | DFP | | |  | GYB | | |
| --- | --- | --- | --- | --- | --- | --- | --- |
| Year | sample size | number of pellets | sum MNI |  | sample size | number of pellets | sum MNI |
| 2000 | 19 | 355 | 777 |  | 14 | 121 | 564 |
| 2001 | 22 | 714 | 2006 |  | 7 | 15 | 183 |
| 2002 | 23 | 556 | 1282 |  | 11 | 36 | 254 |
| 2003 | 8 | 146 | 456 |  | 5 | 47 | 272 |
| 2004 | 21 | 364 | 811 |  | 4 | 65 | 167 |
| 2005 | 19 | 400 | 904 |  | 11 | 194 | 523 |
| 2006 | 15 | 445 | 1095 |  | 16 | 264 | 1016 |
| 2007 | 15 | 465 | 862 |  | 12 | 195 | 469 |
| 2008 | 18 | 410 | 771 |  | 8 | 89 | 244 |
| 2009 | 23 | 495 | 1038 |  | 9 | 297 | 1491 |
| 2010 | 16 | 369 | 683 |  | 3 | 72 | 576 |
| 2011 | 20 | 464 | 1258 |  | 9 | 172 | 600 |
| 2012 | 12 | 314 | 982 |  | 12 | 275 | 1034 |
| 2013 | 5 | 142 | 362 |  | 5 | 81 | 348 |
| 2014 | 5 | 157 | 532 |  | 0 | 0 | 0 |
| 2015 | 17 | 583 | 1316 |  | 8 | 240 | 701 |
| 2016 | 23 | 550 | 963 |  | 1 | 19 | 57 |
| 2017 | 31 | 620 | 1185 |  | 13 | 1061 | 5401 |
| 2018 | 16 | 421 | 848 |  | 10 | 175 | 523 |
| 2019 | 13 | 231 | 524 |  | 8 | 191 | 595 |
| 2020 | 3 | 62 | 140 |  | 6 | 105 | 633 |
| 2021 | 14 | 227 | 676 |  | 7 | 104 | 641 |
| 2022 | 8 | 169 | 371 |  | 6 | 180 | 526 |
| 2023 | 19 | 183 | 404 |  | 10 | 338 | 880 |
| Total | 385 | 8842 | 20246 |  | 195 | 4336 | 17698 |
